# Extending the MaqFACS to measure facial movement in Japanese macaques (*Macaca fuscata*) reveals a wide repertoire potential

**DOI:** 10.1371/journal.pone.0245117

**Published:** 2021-01-07

**Authors:** Catia Correia-Caeiro, Kathryn Holmes, Takako Miyabe-Nishiwaki

**Affiliations:** 1 Primate Research Institute, Kyoto University, Inuyama, Aichi, Japan; 2 School of Psychology, University of Lincoln, Lincoln, Lincolnshire, United Kingdom; CEA, FRANCE

## Abstract

Facial expressions are complex and subtle signals, central for communication and emotion in social mammals. Traditionally, facial expressions have been classified as a whole, disregarding small but relevant differences in displays. Even with the same morphological configuration different information can be conveyed depending on the species. Due to a hardwired processing of faces in the human brain, humans are quick to attribute emotion, but have difficulty in registering facial movement units. The well-known human FACS (Facial Action Coding System) is the gold standard for objectively measuring facial expressions, and can be adapted through anatomical investigation and functional homologies for cross-species systematic comparisons. Here we aimed at developing a FACS for Japanese macaques, following established FACS methodology: first, we considered the species’ muscular facial plan; second, we ascertained functional homologies with other primate species; and finally, we categorised each independent facial movement into Action Units (AUs). Due to similarities in the rhesus and Japanese macaques’ facial musculature, the MaqFACS (previously developed for rhesus macaques) was used as a basis to extend the FACS tool to Japanese macaques, while highlighting the morphological and appearance changes differences between the two species. We documented 19 AUs, 15 Action Descriptors (ADs) and 3 Ear Action Units (EAUs) in Japanese macaques, with all movements of MaqFACS found in Japanese macaques. New movements were also observed, indicating a slightly larger repertoire than in rhesus or Barbary macaques. Our work reported here of the MaqFACS extension for Japanese macaques, when used together with the MaqFACS, comprises a valuable objective tool for the systematic and standardised analysis of facial expressions in Japanese macaques. The MaqFACS extension for Japanese macaques will now allow the investigation of the evolution of communication and emotion in primates, as well as contribute to improving the welfare of individuals, particularly in captivity and laboratory settings.

## Introduction

Facial expressions have been a topic of interest since Darwin’s [1] observations of emotional continuity between species, but only in 1978 [2], a comprehensive and objective system was published to study human facial movement from an anatomical perspective. Using an objective coding system is particularly relevant since faces are processed in an automatic and holistic way in the human brain [3], i.e., people perceive emotion and meaning easily on faces (both human and non-human), but are not proficient when identifying the subtle and independent behaviours of the face (e.g. brow raising). Seiler [4] summarised this problem a few years before FACS (Facial Action Coding System, from here on, HumanFACS) was published: "The human eye observing facial expressions in monkeys or apes is not trained to notice all the movements and twitches (…) and the observer finds it difficult to objectify what he observes". Furthermore, investigating facial expressions, particularly in animals, may be subject to a wide range of serious issues that are common both in research and in human-animal interactions: 1) anthropomorphism, in which people naturally interpret animal behaviour as if the individuals were human (e.g. the misattribution of "guilt" to a dog facial expression when scolded, instead of recognising the fear response [5]); 2) difficulty in inter-species comparison, both due to using too broad concepts making comparisons difficult (e.g. "grimacing" [6]), but also due to simplistically applying the same knowledge to different species (e.g. [6]); 3) subjective assessments between coders (i.e. when behaviours are described with broad words, it is harder for different coders to agree, for example coding "happy face" is very subjective and can be affected by a range of factors [7]); 4) contextual *a priori* assumptions, where evaluators use contextual cues to ascribe valence/emotion to a behaviour (e.g. the dog "feels guilty" because they did something forbidden [5]); 5) appearance-biases (e.g. natural wrinkles perceived as movement [8]), and morphology-meaning errors, when having the same basic facial configuration (e.g. teeth exposed) in two different species (e.g. rhesus macaques vs. humans) is conflated with having the same meaning (when it actually doesn’t: in macaques signals submission [9], in humans it conveys a greeting or a positive emotion [10], and is highly context-dependent [9, 10]). The use of HumanFACS, the gold standard for the research of human facial expressions, used for over 40 years, avoids all these problems by allowing: 1) objective quantification of detailed and independent facial movements on any species where the musculature is known; 2) systematic and standardised coding, 3) reliable coding through training and certification of coders, and 4) cross-species comparison through muscular homologies. Due to all the sources of biases mentioned above, FACS is considered the more objective, and is also the more standardised method between species in comparison with alternative methods (e.g. categorisation- or emotion-based) to study facial behaviour [11].

In the last decade, researchers have been adapting FACS to several species, including non-human primates: chimpanzees [12], orangutans [13], rhesus [14], Barbary [15] and crested macaques [16], gibbons and siamangs [17], and domestic species: dogs [18], cats [19], and horses [20]. The development of FACS for other species has, for example, revealed unique aspects of cognition in orangutans using OrangFACS (i.e. play faces are intentional [21]) and chimpanzees using ChimpFACS (i.e. facial expressions can be categorised [22]), as well as functional aspects of communication (i.e. facial expression length is context-dependent [23]). Researchers have also used the DogFACS alongside HumanFACS to investigate how these two unrelated species that have lived together and are thought to have coevolved social cognition [24], are able to influence [18] and perceive each other [25]. Furthermore, because animal FACS are built within a comparative framework, it is possible to compare facial repertoires across species; a comparison of EquiFACS and ChimpFACS demonstrated that horses have a larger potentiality for movement than chimpanzees [20]. Similarly, a comparison of different species of hylobatids concluded they have some degree of control over their facial expressions and direct them mostly to social partners [23]. These studies demonstrate that only by using FACS as a tool was it possible to investigate different species in parallel and a range of specific questions in such an objective and systematic way. A review of multi-species FACS identified three main types of applications [11]: 1) building the phylogeny of facial behaviours between humans and other animals in order to understand how they evolved, 2) understanding the cognitive mechanisms within facial communication, and 3) socio-ecological factors that shape facial behaviours. Other researchers [26–28] have suggested the use of facial expressions as an indicator of welfare (positive and negative), pain, emotion and intent. In these contexts, where biases are even harder to be avoided due to expectation biases in observers [29] or interpretative anthropomorphism [7], using an objective system like FACS is paramount.

Extending FACS to Japanese macaques will allow for expansion of research into this species’ expression and communicative abilities, as well as facilitating comparative studies within the genus *Macaca* and other non-human primates. In particular, the genus *Macaca* has been extensively studied due to their wide variation of morphology and behaviour between and within the four monophyletic taxa [30, 31]. Other than sharing some basic features (e.g. semi-terrestriality or multi-male multi-female grouping), the 23 species of macaques have been suggested to present the greatest interspecies variation in primates within a single genus [31]. This is particularly highlighted by the wide variation of patterns of behaviour in macaque species (e.g. reconciliation, temperament) and gradation in social organisation (e.g. from very tolerant to very nepotistic) [31]. This variation has also been reported for non-vocal communication in macaques. Initial broad qualitative descriptions of facial expressions and gestures in macaques [32, 33] reported lower interspecific variation in agonistic displays than in affiliative displays, while more recent studies [11] have highlighted that the size and the characteristics of the non-verbal cues, along with conciliatory and counter-aggression differences between phyletic groups are important to consider in order to understand the evolution and function of the *Macaca* communication.

**Fig 1:**
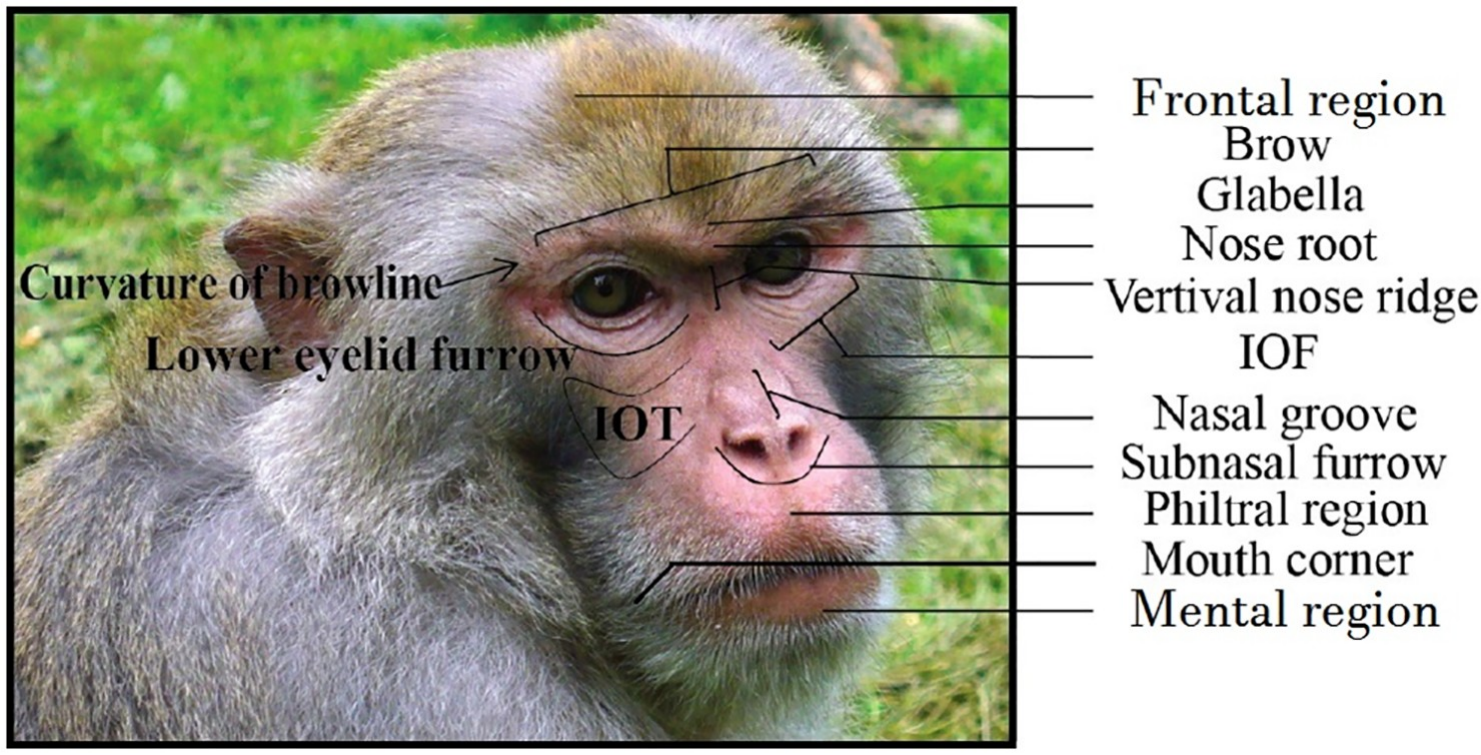
Facial landmarks in rhesus macaques (adapted from [15]). IOT—Infraorbital triangle, IOF—Infraorbital furrow.

**Fig 2:**
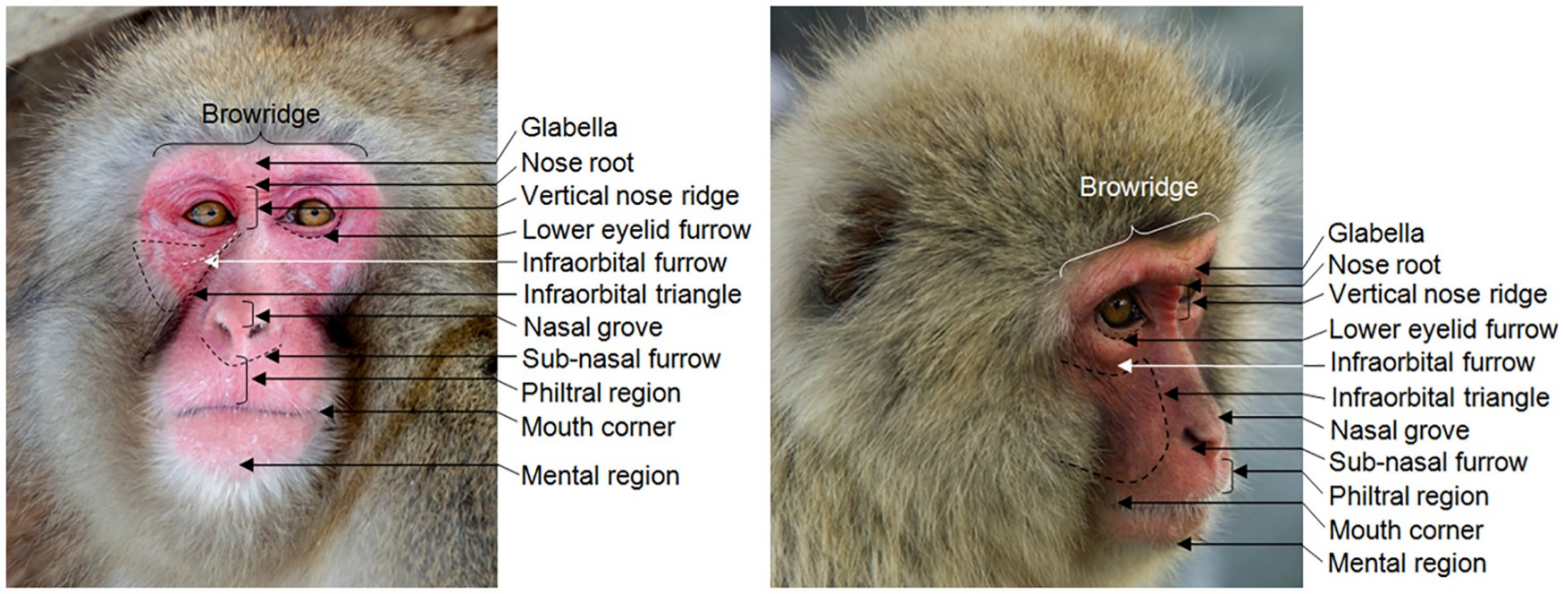
Facial landmarks in Japanese macaques in frontal and profile view. The Japanese macaque has a longer and more prognathic face than rhesus macaques, with lower intra-ocular distance. The ears tend to be more covered with hair. In some individuals, the browridge may be more salient laterally.

Due to the wide morphological variation in the *Macaca* genus, it is important to consider differences and similarities between species in order to create a new FACS, and in particular, how the target species (*Macaca fuscata*) differs from the other species. A rhesus macaque-like population ancestral to the Japanese macaque migrated between 0.43–0.63 Ma ago from the Korean peninsula to the Japanese islands [34–36], adapting its ecological and life history patterns to the new habitat. Differences in morphology have also been reported between extant rhesus macaques and Japanese macaques, including on the face, with the latter presenting higher and more prognathic faces, with lower intra-ocular distance. Morphological facial differences may affect facial movement classification, particularly changes in facial landmarks (Figs 1 and 2), so they must be investigated and incorporated into FACS development.

Despite their differences, rhesus and Japanese macaques display a similar group size [37, 38] and social style [39], both factors linked to communication facial patterns [40–42]. Both species are more despotic than other macaques, with mainly unidirectional conflicts, reduced conciliation (more often between kin), and a rigid dominance hierarchy, where social interactions are kin-biased [39, 43, 44]. Due to the more despotic social style [39], Japanese macaques’ facial expressions are predicted to form a relatively small repertoire. However, the opposite might also be true, since they still form large groups and are the largest macaque from the genus with larger faces, which is linked to a larger facial repertoire and improved visual acuity [41, 45]. Hence, developing FACS for this species will enable precise quantification of facial expression repertoires in the Japanese macaque in future studies.

Regarding facial expressions in Japanese macaques, only a limited number of studies have been published. An experimental study [6] used only seven broad based categories (e.g. grimace, gaze aversion) to test the influence of a variety of factors on facial expressions in Japanese macaques. These categories were not specific to Japanese macaques, but were based on Redican’s book [46] that discusses all primate species’ facial expressions as a whole, which might have limited the conclusions achieved (e.g. most differences were seen in males). Before animal FACS were developed, Kanazawa [47] noted that FACS was the most standardised tool for human facial expressions, but without knowing how FACS could be translated to macaques, the author not only questioned previous results investigating facial expressions, but also stated that it was very difficult to identify critical facial movements. This might be why studies of Japanese macaque behaviour usually have very few facial behaviours included [48–50] even though face-to-face engagement is important in Japanese macaque social interactions [51, 52].

Adapting FACS for Japanese macaques will not only allow a more detailed analysis of individual facial movements, but will be particularly important for their welfare assessment (e.g. through potential facial movements associated with fear or pain, see [26, 53] for reviews on the topic of pain assessment through facial expression in animals) of captive and laboratory individuals. Although no studies have been published yet for Japanese macaque pain facial expressions, two studies reported facial changes related to pain in other macaque species. One study observed "flinching" of eye and back of the head muscles when pain was induced in the crab-eating macaque, and used these as a measure of pain [54, 55]. Another study reported lip tightening and chewing in rhesus macaques after surgery [53]. The assessment of pain in Japanese macaques might be particularly important, since this species is the third most used in laboratory research in Japan due to their reputation as more intelligent, gentle and trainable than rhesus and crab-eating macaques, as well as due to their homogenous genetic background [56]. In neuroscience targeting the higher brain functions, it is thought to be the preferred species, typically using invasive procedures [56]. The human FACS has been used extensively to study human facial expressions of pain (e.g. [57]), and when extended for Japanese macaques, it will have the potential to be used to create a welfare tool to assess facial movement potentially associated with pain in this species [26, 53].

The main goal of this study was to fill a gap in the literature by developing a new tool for measuring facial behaviour in Japanese macaques, in an objective, standardised and systematic way. Since FACS is the gold standard for objective measurement of facial behaviour, we followed the same methodological steps that characterise the development of these tools and that have served as basis for all previous animal FACS adaptations. We first considered the facial musculature of Japanese macaques, then ascertained the function of each individual muscle, and finally categorised and described each individual facial movement observed. As with all previous FACS, the FACS for Japanese macaques will be a freely available tool with potential applications in a variety of areas, ranging from fundamental questions within the evolution and function of communication and emotion in primates and humans, to applied work on animal welfare.

## Methodology

### Subjects and data collection

This study follows the Guideline of Care and Use of Nonhuman Primates, KUPRI and was approved by the Animal Welfare and Care Committee of KUPRI (2019–165). All work undertaken for this manuscript was purely observational.

For the purpose of adapting FACS for Japanese macaques, we recorded spontaneous behaviour from Japanese macaques (*Macaca fuscata fuscata*) at two locations in Japan: in two outdoor enclosures at the Primate Research Institute, Kyoto University (KUPRI), housing 89 Japanese macaques (54 females, age range: 1-22yo), and at Koshima deserted island, which is inhabited by two troops of approximately 160 Japanese macaques. The individuals at the KUPRI outdoor areas (729–960 m^2^) were grouped by their region of origin (37 from Wakasa and 52 from Takahama). Each enclosure had access to indoor heated quarters, and featured varied climbing structures and environmental enrichment objects, such as platforms, ropes, swings, wooden toys, and feeders. Individuals had visual, olfactory and auditory contact between the two groups, as well with other animal species from the surrounding area (e.g. crows). Macaques were fed twice daily (once in the weekends) during the morning and afternoon with monkey chow, wheat and sweet potatoes, and occasional fresh tree branches. Water was supplied ad libitum. Please see [58] for more details on KUPRI individuals. The individuals at Koshima are free-ranging, were habituated to humans, and have been partly provisioned and monitored by researchers since 1952 [59]. See [59, 60] for detailed information on Koshima individuals.

We recorded approximately 7h of video at KUPRI and 13h at Koshima, including ad libitum behaviour and opportunistic focals featuring any behaviour (including solitary, conspecific, agonistic, affiliative, resting, grooming, foraging/feeding, sexual, play, and human interaction behaviours, among others), and during varied observational field experiments (see S1 Text for more details). Additionally, approximately 1h of video clips were selected from YouTube (marked as CC copyright or with author permission), mainly featuring individuals from Jigokudani and Hagachizaki Monkey Parks in a few additional contexts (e.g. hot spring bathing, snow foraging, etc.). The videos collected were comprised of close-up faces in a wide variety of behaviours and contexts, in a large sample of individuals, in order to try to document the *full potential* of facial movement in this species (as opposed to the *typical* movements of the species, which can only be achieved once the FACS for Japanese macaques is published and using a different sample). The collection of videos used here totalled 21h and over 250 individuals from five distinct populations, which was enough to identify at least two examples of each facial movement (see next section for definition of facial movement) produced by each muscle of the Japanese macaque face (see S1 Text for rational for target sample). It is still possible that rare movements might have not been observed (e.g. from contexts we did not sample, such as chronic pain), but as with previous animal FACS, if additional movements are later observed and published, it is possible to add them through the dedicated cross-species FACS website (www.animalFACS.com).

### Applying MaqFACS to Japanese macaques

Normally, in *Step 1* of a FACS adaption, the anatomical plan for the species is built through either performing dissections in individuals of the target species (e.g. [18]) and/or already published dissections (e.g. [13]). This is accompanied by noting the differences in facial musculature and ascertaining functional homologies between the target species, humans and other closely related species. However, the target species of the current FACS adaptation, Japanese macaques, and rhesus macaques, are reported to have identical facial musculature (in regards to presence of individual muscles and its relative position on the face) [61], which is not surprising given that these are the most closely related of the genus *Macaca*. Seiler [4] noted only one difference between *M*. *mulatta* and *M*. *fuscata* muscles in the temporal region, where in the latter the fibres of the auricularis anterior are longer than in the former, running parallel to the orbito-temporalis so that both muscles seem to be one. Even when comparing facial muscles of *Macaca nigra* and *M*. *mulatta*, which are from different evolutionary lineages of macaques [62], the former was only missing 4 out of 24 muscles present the latter [61], suggesting facial musculature in the genus to be well conserved. Hence, given that Japanese and rhesus macaques are reported to have high similarity in facial musculature, and rhesus macaques have already a dedicated FACS (i.e. the MaqFACS [14]) developed based on information from dissections of their facial musculature [63], we instead can test the application of the MaqFACS [14] for Japanese macaques. As the FACS codes are based on the facial musculature of a species, if two species have identical anatomical plans, then the same FACS can be applied to both species. Nonetheless, these two species still have facial morphological differences, and therefore we need to verify if the MaqFACS can be applied to Japanese macaques (see *Step 3* below).

In *Step 2* of FACS adaptations, intra-muscular electrical stimulation is usually employed to verify the link between muscle contractions and appearance changes [64, 65]. However, due to anatomical similarity to *M*. *mulatta*, combined with ethical concerns, this step was not performed for the Japanese macaque. The omission of this step has been done in most animal FACS adaptations for similar reasons (e.g. [13, 15, 17]).

*Step 3* usually comprises of frame-by-frame video observation of spontaneous facial behaviours to identify independent facial movements, along with a list of appearance changes based on facial landmarks (Fig 2), and the minimum coding criteria for each movement. These movements are then linked to the underlying musculature through functional homology. For Japanese macaques, this step was performed using the MaqFACS to identify the movements already described for rhesus macaques, while simultaneously noting morphological differences between the appearance changes of the two species. Additionally, we looked for possible movements not included in the MaqFACS using the functional homologies of human facial muscles. In the FACS manuals, each movement is linked to the contraction of a particular mimetic muscle and is designated as Action Unit (AU) or Ear Action Unit (EAU), for face and ears respectively, and given a numerical code with a descriptive designation, as per previous FACS nomenclature (e.g. the lip corners are pulled backwards by the zygomaticus major muscle, which is designated as AU12—Lip corner puller). Action Descriptors (ADs) produced by non-mimetic muscles are also identified, since these can impact and modify appearance changes in AUs. All movements are accompanied by video examples (see S1 Text), in real-time and in slow-motion, whenever movements are too fast to be watched in real-time.

Importantly, the information here reported for Japanese macaques must be used as an additional resource to the MaqFACS, and does not replace training and certification in MaqFACS. This work thus aims at being used as a *MaqFACS Extension for Japanese macaques*, supported by video examples for this species. This approach was used before in two studies [15, 16], where authors reported that the MaqFACS developed for rhesus macaques [14] could be used with Barbary (*Macaca sylvanus*) and crested macaques (*M*. *nigra*), which are phylogenetically more distant from rhesus macaques than Japanese macaques [30, 62].

### Coding reliability

We tested inter-observer reliability (between CCC, certified in several FACS systems, including MaqFACS and HumanFACS, and KH, certified in MaqFACS) by coding an additional seven clips totalling 24min (x̄ = 3.43min) of pre-existent footage with different individuals from the ones described in "Subjects and data collection". This footage had been collected beforehand for another research project during feeding and training tasks, focusing on the face of 21 Japanese macaques (14 females, age range: 7–22, x̄ = 11.5yo), living indoors in pairs/trios at KUPRI. The inter-reliability coding had a three-fold aim: 1) to verify which MaqFACS movements could be applied to Japanese macaques and if the new movements found in Japanese macaques could be coded consistently; 2) to check if both coders were able to reliably identify the MaqFACS movements in Japanese macaques while accounting for potential morphological differences between rhesus and Japanese macaques, 3) if agreement was low for particular AUs, modify appearance changes descriptions to improve identification of AUs between coders, followed by further rounds of coding, alternated with improvement in appearance changes description.

The coder’s overall reliability (Wexler’s index [66], see Eq (1)) and the AUs independent coding agreement (calculated through the average of each AU agreement) from a first round of coding (Table 1) indicated a low overall agreement between coders of 53%. After comparing both coders work, it was revealed that this low agreement stemmed mostly from minor differences in coding ADs due to coders technical assumptions (e.g. KH coded all individual masticatory movements such as AU25, AU26, etc., whilst CCC coded only AD81—Chewing during mastication, as recommended for the HumanFACS, [67, 68], since these masticatory movements are not produced by mimetic muscles). Hence, CCC recoded the mouth movements to account for this, which increased the overall reliability to 81% (Table 1), which is considered a good agreement [12, 68]. However, some AUs still had low independent agreement, particularly in movements derived from mimetic muscles (e.g. AU1+2), which were discussed between the coders to flag what lead to such differences. This was then followed by a third coding round by both coders, which helped clarify the description of appearance changes for some movements and improved reliability. In this third round of coding, we obtained a mean of 89% overall agreement on Wexler’s index (1) [66], and also a good agreement on most AUs (Table 1).

$$Wexler\prime s\;index=\;\frac{(Number\;of\;AUs\;on\;which\;coder\;1\;and\;Coder\;2\;agreed)\times 2}{The\;total\;number\;of\;AUs\;scored\;by\;the\;two\;coders}$$ (1)

**Table 1:** Wexler’s index (1972) and independent coding agreement for each AU, AD and EAD in three coding rounds.

	Round 1	Round 2	Round 3
Wexler’s index	0.53	0.81	0.89
AU1+2	42.50	43.73	88.27
AU41	18.25	19.07	76.06
AU43	0.00	0.00	78.57
AU45	80.98	84.09	88.47
AU10	42.64	39.41	63.06
AU9+10	27.08	37.50	64.29
AU12	43.37	58.97	73.72
AU16	14.12	61.83	64.24
AU17	58.35	86.00	91.02
AU18i	0.00	13.33	65.82
AU18ii	22.22	10.00	76.19
AU24	32.02	64.58	69.84
AU25	28.97	87.06	86.99
AU26	53.60	73.13	75.43
AU27	17.68	67.70	73.12
AU8	2.82	78.36	78.52
AU38	25.00	25.00	50.00
AD181	25.00	16.67	83.27
AD19	88.75	96.92	92.15
AD119	4.17	73.61	70.24
AD29	0.00	37.50	45.92
AD30	24.00	64.00	77.38
AD32	0.00	0.00	42.86
AD33	0.00	50.00	85.71
AD34	0.00	0.00	71.43
AD35	0.00	18.75	52.04
AD36	0.00	23.33	63.10
AD80	0.00	60.00	76.67
AD81	39.71	51.99	73.54
AD86	8.33	16.67	85.03
AD160	33.33	33.33	90.48
AD100	0.00	18.69	80.84
EAD1	0.00	0.00	61.90
EAD2	0.00	0.00	57.14
EAD3	13.10	14.68	71.43

Note: AU6 was not observed in these videos, but both coders agreed in all rounds it was not present. We observed AU6 in other videos without any difference to note from rhesus macaques (see Results).

## Results and discussion

Since our results are to be used as an extension from the MaqFACS, we report here only differences in appearance changes between rhesus and Japanese macaques for each AU, as well as new movements we observed in Japanese macaques, not described in the MaqFACS. Therefore, we strongly recommend the MaqFACS extension to always be used in conjunction with the original MaqFACS manual and only after certification in MaqFACS for rhesus macaques. In Table 2, we compile the previously published information on the presence/absence of Action Units and its underlying facial muscles for humans, rhesus macaques and Barbary macaques in comparison with what we found for Japanese macaques. Both the information from Table 2 and the AUs descriptions that follow, result from the following points: a) Application of the MaqFACS to Japanese macaques footage to find examples of each AU, while noting morphological differences in appearance changes; b) Identification of new movements in Japanese macaques (not previously included in the MaqFACS); and c) Using MaqFACS together with the MaqFACS extension for Japanese macaques, i.e., the information generated by point a) and point b), verify if the AUs from MaqFACS and its extension can be reliably coded in Japanese macaques, while improving the description of each AU for Japanese macaques.

**Table 2:** Comparison between the Action Units (AUs) and Ear Action Units (EAUs) previously included in the FACS for humans [67], rhesus macaques [14], and Barbary macaques [15] with what we identified here for Japanese macaques.

AU code	AU name	Underlying muscle	Human	Rhesus macaque	Barbary macaque	Japanese macaque
**AU1**	**Inner Brow Raiser**	Frontalis (medial)	✓	**x**	**x**	**x**
**AU2**	**Outer Brow Raiser**	Frontalis (lateral)	✓	**x**	**x**	**x**
**AU1+2**	**Brow Raiser**	Frontalis	**x**	✓	✓	✓
					+AU1+2 unilateral	+AU1+2 unilateral
**AU4**	**Brow Lowerer**	Procerus, Depressor supercilii, Corrugator supercilii	✓	**x**	**x**	**x**
**AU41**	**Glabella Lowerer**	Procerus	✓	✓	✓	✓
**AU5**	**Upper Lid Raiser**	Orbicularis oculi	✓	**x**	**x**	**x**
**AU6**	**Cheek Raiser**	Orbicularis oculi, pars orbitalis	✓	✓	✓	✓
**AU7**	**Lid Tightener**	Orbicularis oculi, pars palpebralis	✓	**x**	**x**	**x**
**AU43**	**Eye closure**		✓	✓	✓	✓
**AU45**	**Blink**		✓	✓	✓	✓
**AU8**	**Lips Towards Each Other**	Orbicularis oris	✓	✓	✓	✓
**AU9**	**Nose Wrinkler**	Levator labii superioris alaeque nasi	✓	**x**	**x**	✓^2^
**AU10**	**Upper Lip Raiser**	Levator labii superioris	✓	✓	✓	✓
**AU9+10**	**Nose Wrinkler and Upper Lip Raiser**	LLSAN, LLS	**x**	✓	✓	✓
**AU11**	**Nasiolabial Furrow Deepener**	Zygomatic minor	✓	**x**	**x**	**x**
**AU12**	**Lip Corner Puller**	Zygomatic major	✓	✓	✓	✓
**AU13**	**Cheek Puffer**	Caninus (= levator anguli oris)	✓	**x**	**x**	**x**
**AU14**	**Dimpler**	Buccinatorius	✓	**x**	**x**	**x**
**AU15**	**Lip Corner Depressor**	Depressor anguli oris	✓	**x**	**x**	**x**
**AU16**	**Lower Lip Depressor**	Depressor labii	✓	✓	✓	✓
**AU17**	**Chin Raiser**	Mentalis	✓	✓	✓	✓
**AU18**	**Lip Pucker**	Incisivii labii (superioris and inf.)	✓	**x**	**x**	**x**
**AU18i**	**True Pucker**	Orbicularis oris, incisivii labii	**x**	✓	✓	✓
**AU18ii**	**Outer Pucker**	Incisivii labii	**x**	✓	✓^1^	✓
**AU20**	**Lip Stretcher**	Risorius	✓	**x**	**x**	**x**
**AU21**	**Neck Tightener**	Platysma myoides	✓	**x**	**x**	**x**
**AU22**	**Lip Funneler**	Orbicularis oris	✓	**x**	**x**	**x**
**AU23**	**Lip Tightener**		✓	**x**	**x**	**x**
**AU24**	**Lip Presser**		✓	**x**	**x**	✓
**AU25**	**Lips Parted**	Orbicularis oris, levator labii superioris, depressor labii inferioris, non-mimetic m.	✓	✓	✓	✓
**AU26**	**Jaw Drop**		✓	✓	✓	✓
**AU27**	**Mouth Stretch**		✓	✓	✓	✓
**AU28**	**Lip Suck**	Orbicularis oris	✓	**x**	**x**	✓
**AU38**	**Nostril Dilator**	Nasalis	✓	**x**	**x**	✓
**AU39**	**Nostril Compressor**	Nasalis, Depressor septi nasi	✓	**x**	**x**	**x**
**EAU1**	**Ears Forward**	Anterior auricularis	**x**	✓	✓	✓
**EAU2**	**Ears Elevator**	Superior auricularis	**x**	✓	✓	✓
**EAU3**	**Ears Flattener**	Posterior auricularis	**x**	✓	✓	✓

^1^Present, but appears to be rare in Barbary macaques.

^2^Only one observation in Japanese macaques.

The following sections report the differences between what was described for rhesus macaques in the MaqFACS and what we observed for Japanese macaques in each AU, and may include the following points: 1) comparative muscular basis (for Japanese and rhesus macaques this information is taken from the MaqFACS, but given in a comparative perspective); 2) main appearance changes description (for full list of appearance changes, please refer to the MaqFACS manual), 3) important differences to consider during coding between Japanese macaques and other primate species (mostly rhesus and Barbary macaques, and humans). As such, this MaqFACS extension is to be used *in addition to* the MaqFACS manual. In some cases, AUs subsections are preceded by a section noting the main morphological and anatomical differences on the static/neutral face (e.g. facial landmarks) for a particular region (e.g. Upper face), as it can impact coding of the dynamic states of the face.

### Upper face Action Units

**Fig 3:**
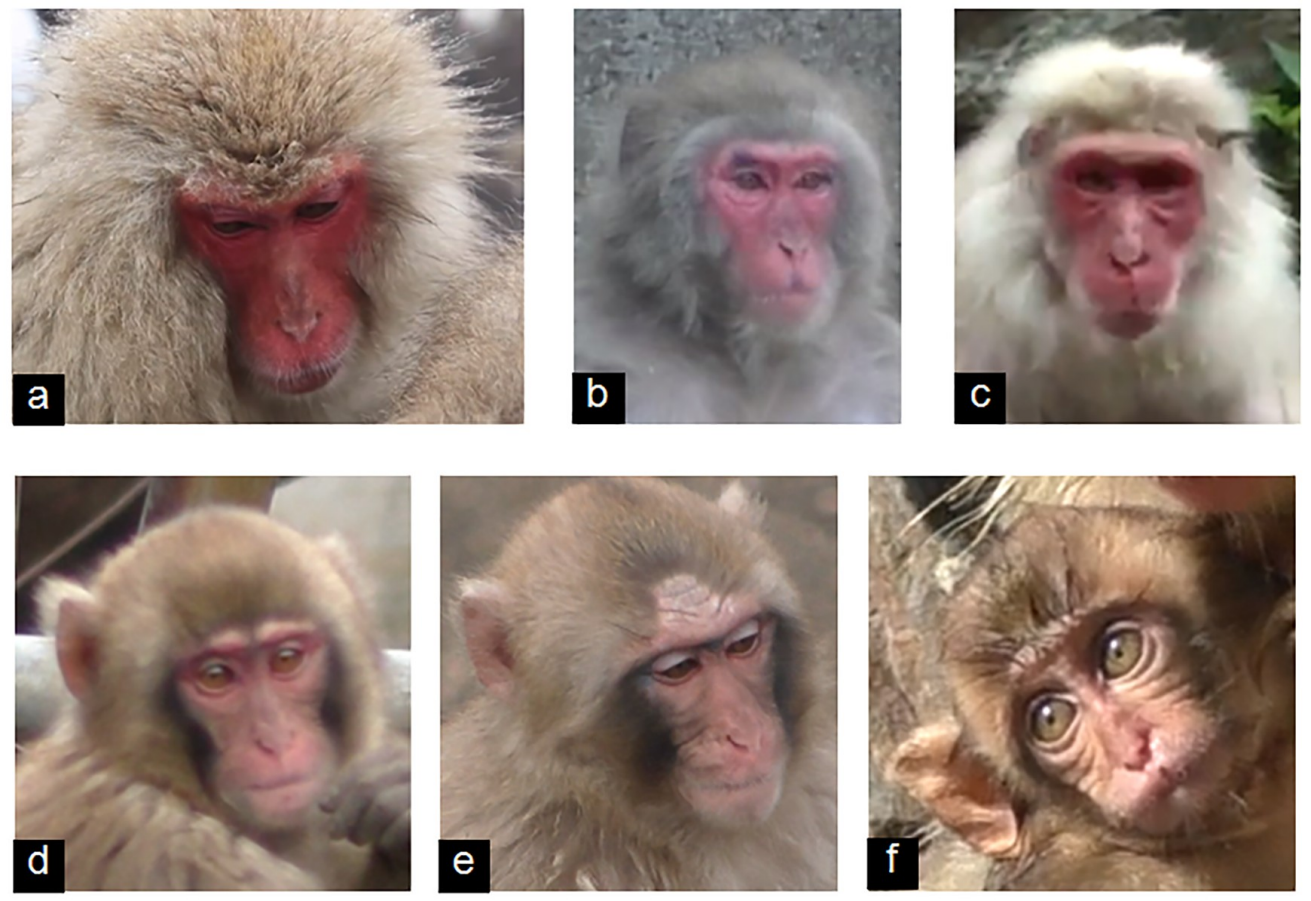
Browridge shape and size diversity in Japanese macaques adults (a–c) and infants (d–f).

While the Japanese macaques’ browridge is not as pronounced as in apes (e.g. chimpanzee), it is more salient than in humans, presenting cranio-caudal movement. The superior surface of the browridge is usually covered in hair similar to the body and head hair, but slightly longer (Fig 3b). In some individuals the hair covering the browridge may vary (Fig 3). The movement of the hair in this region is important as appearance changes for the AUs that move the brows (i.e. AU1+2 and AU41, see below), as the hair attached to the browridge moves independently of the hair attached to the frontal region and aids in the AU identification. In some individuals, a slight gap (Fig 3a) can be observed between the browridge hair and the frontal region hair. In young infants, the browridge is not fully developed as in adults, being less salient and presenting two half browridges (Fig 3b–3d).

**Fig 4:**
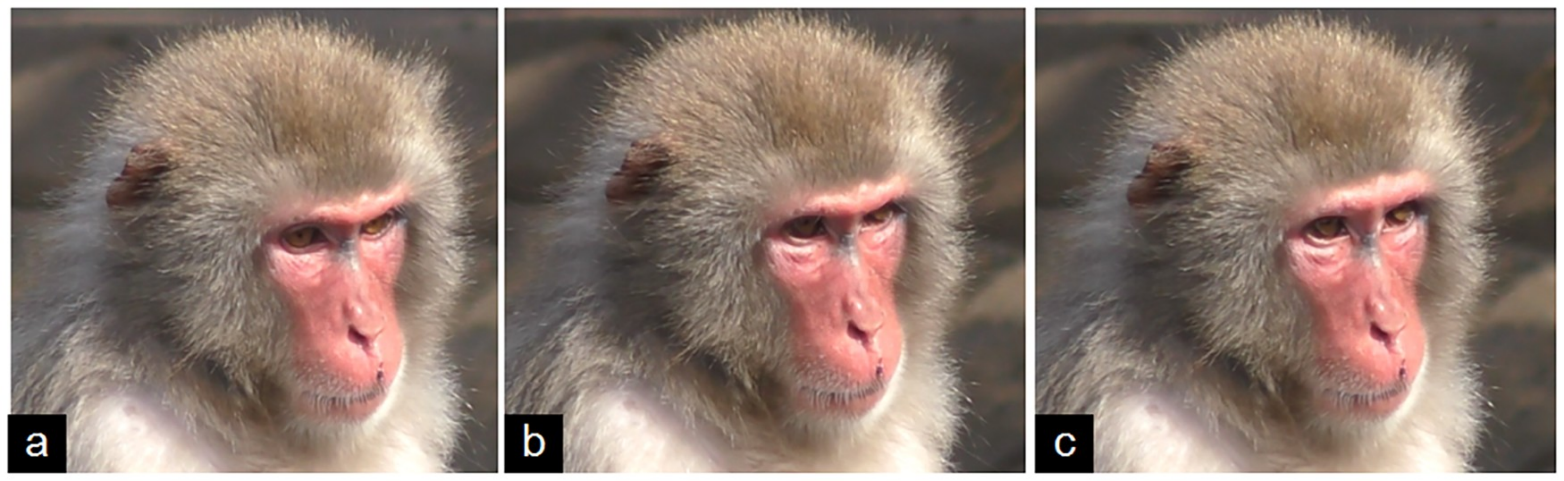
**a**—Neutral face; **b**—AD60—Eyes up is acting, with the pupil moved upwards; **c**—In the next frame, AU1+2—Brow raiser is added to AD60.

Brow movements have been reported to be associated to some extent to eye movements, as when individuals look up, down or even left/right, the frontalis and depressor supercilii muscles are often activated [4], raising or lowering the brow. This is what we observed here as well, although eye movements were also recorded independently from the brow movements or with a clear different temporal onset (e.g. Fig 4, S1a and S1b Video).

Individuals’ browridges were regularly moving, often coupled with eye movements, which may make it challenging to determine the neutral position of the browridge for each individual, particularly due to individual variation in, for example, hair covering the skin or protuberance of this region. The angle of recording or head movements can also influence judgement on the position of the browridge, and therefore it is advisable to code these movements in video only, after determining first the neutral brow position for each individual, and account for the position of the camera relative to the individual’s face, while avoiding coding stills on their own (except for higher intensity AU1+2 or AU41 where appearance changes are easier to identify).

We identified 5 independent movements in the upper face of Japanese macaques, AU1+2, AU41, AU43, AU45 and AU6, which are described below and differences noted with humans and rhesus macaques.

#### AU1+2—Brow raiser

1) Comparative muscular basis: In humans, AU1 (Inner brow raiser) and AU2 (Outer brow raiser) are independently produced by the contraction of the medial frontalis and the lateral frontalis, respectively. In rhesus macaques, the medial and lateral frontalis muscles also contract independently when stimulated [65].

2) Main appearance changes: despite independent muscle contraction in rhesus macaques, AU1 and AU2 were not observed in isolation in spontaneous behaviour, and therefore AU1+2 is coded when the browridge is raised. In Japanese macaques, we found the same merged movement, so AU1+2 is coded (S2a and S2b Video). In strong actions of the frontalis, Seiler [4] observed that the ears are always pulled back (EAU3) before the brow is raised. However, our observations of Japanese macaques’ spontaneous behaviour contradicts Seiler’s report, since we documented strong actions of the brow without any ear movement (S3a, S3b and S4 Videos). This difference might be due to Seiler’s particular context of observation.

3) Differences: Additionally, we found a unilateral AU1+2 (AU1+2U), identical to what Julle-Daniere et al. [15] described for Barbary macaques. In AU1+2U, the frontalis produces movement in only one hemiface (S5a, S5b and S6 Videos), or of unequal intensities on each hemiface, producing a slanted browridge. This lateralised contraction of the frontalis was not described in the MaqFACS, but interestingly, it was observed in rhesus macaques during EMG experiments [4] and during intra-muscular electrical stimulation [65].

#### AU41—Glabella lowerer

1) Comparative muscular basis: In humans, the brows are lowered and brought together by the action of three muscles (procerus, depressor supercilii and corrugator supercilii), resulting in the AU4—Brow lowerer. These muscles have also been identified in all the other macaque species here referred.

2) Main appearance changes: Despite the presence of the necessary muscles for AU4 in macaques, AU4 is not observed in Japanese macaques (nor in rhesus or Barbary macaques). In Japanese macaques, only AU41 was observed, where the browridge is brought downwards by the procerus muscle, but there is no corrugation on the glabella (S7a, S7b, S8 and S9 Videos).

3) Differences: Thus, AU4 seems to be observed exclusively in humans, since it brings the eyebrows closer together through the corrugator supercilii m. action, while AU41 only lowers the brows. This movement was described in Japanese macaques [4] to be frequent during dozing, which means individuals may contract this muscle to sleep, to aid in maintaining the eyes closed or to shield from light, which is not observed in humans. It might also be that brow movements in Japanese macaques are part of the awake/attentive/neutral state of the individual, with almost constant low intensity movements both upwards and downwards. This is what we observed in our videos, as it was rare to not see some brow movements in all the contexts observed.

#### AU43/AU45—Eye closure/blink

**Fig 5:**
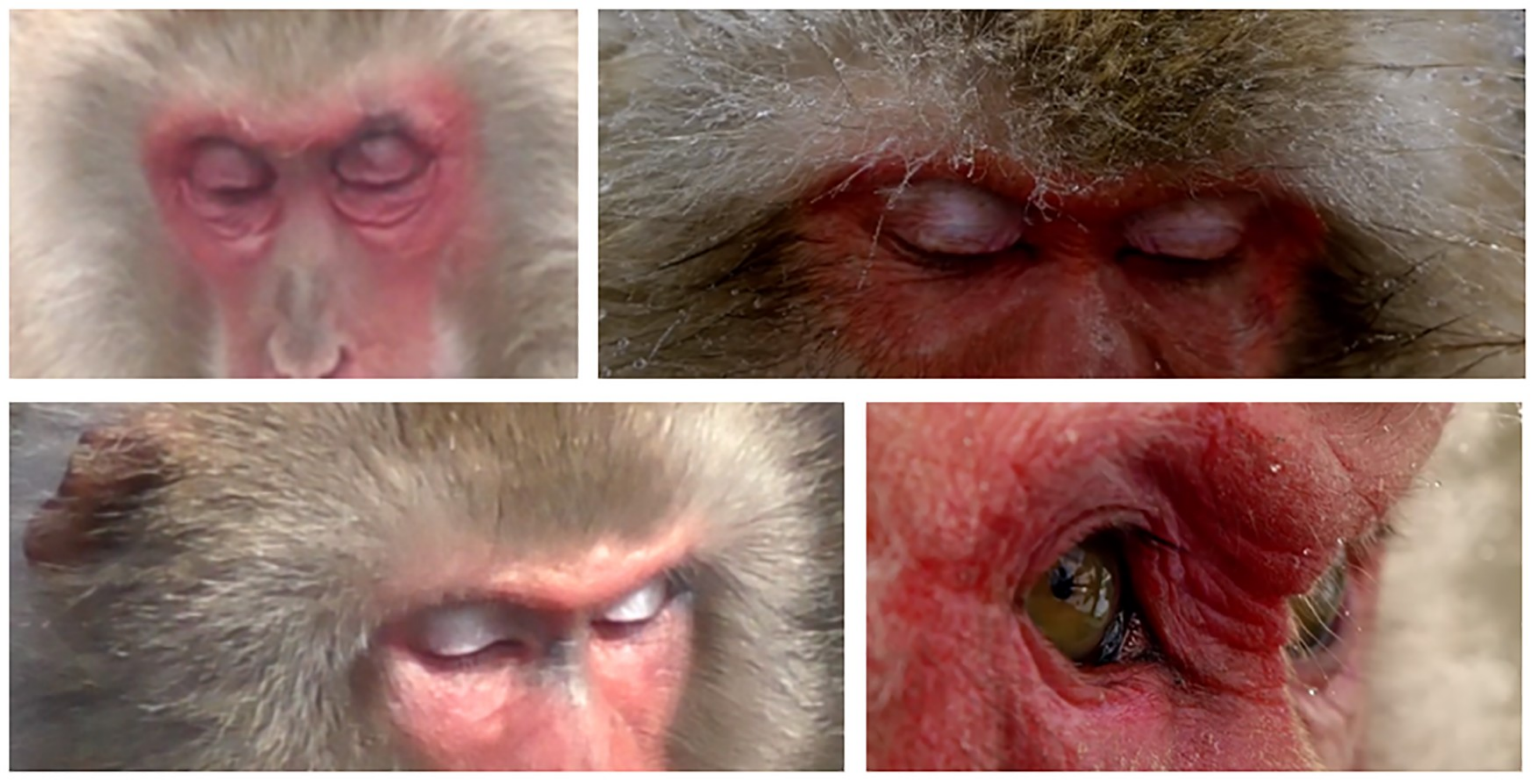
Variation in eye region morphology, including upper eyelid coloration and wrinkling, bulging, bagging differences around the eyes and nose ridge.

1) Comparative muscular basis and 2) Main appearance changes: The eye closure and blink in Japanese macaques seems to recruit to some extent the lower portion of the orbicularis oculi m. often, with the lower eyelid raising slightly or twitching, even in a low intensity blink, where the eyelid does not close completely (recruitment of orbicularis oculi pars palpebralis m., as also described in the MaqFACS). Low intensity movement is visible in the corner of the eyes as well, where the skin both superiorly and inferiorly is slightly pulled towards the eye, and wrinkles slightly or deepens existing wrinkles. The pars palpebralis portion of the orbicularis oculi m. thus must extend slightly outside the eyelids, but this is not however enough to code an AU6—Cheek raiser (see below).

3) Differences: Unlike in humans, where the sclera is very visible and aids in these AUs identification, the sclera in macaques is not visible in neutral forward eyeball position, but when there are eyeball movements, a small portion of white sclera appears laterally. Japanese macaques have a few short black eyelashes on both eyelids and the upper eyelid has a lighter colour in some individuals, sometimes with a white/blue coloration, contrasting with the surrounding darker red/pink of the face. The iris is coloured brown and the pupil is black, visible at close range. There is some morphological variation around the lower eyelid furrow and the infraorbital triangle, with some individuals presenting no wrinkles under the eye, while others present some wrinkles in neutral face, particularly in infants (Fig 5). To note that in the infant face, the facial skin is light pink or cream, with mouth and mental regions lighter, and eyelids white/blue, changing the contrast of the facial skin and surrounding areas.

#### AU6—Cheek raiser (+AU43)

3) Differences: AU6 in humans is easily identified due to the fat deposit on the cheek area. In Japanese macaques (S10a, S10b, S11a and S11b Videos), there is no fat deposit, which changes greatly AU6 appearance changes. However, the AU6 in Japanese macaques is identical to AU6 in rhesus macaques, and was also seen only with AU43. Hence, the appearance changes are the same as per the MaqFACS. Infants have more wrinkles in the IOT (infraorbital triangle) than adults in the neutral face (Fig 3), which can be a false appearance change for AU6. Hence, comparison with infant neutral faces is essential.

### Lower face Action Units

#### AU8—Lips towards each other (+AU18+AU26/AU27)

**Fig 6:**
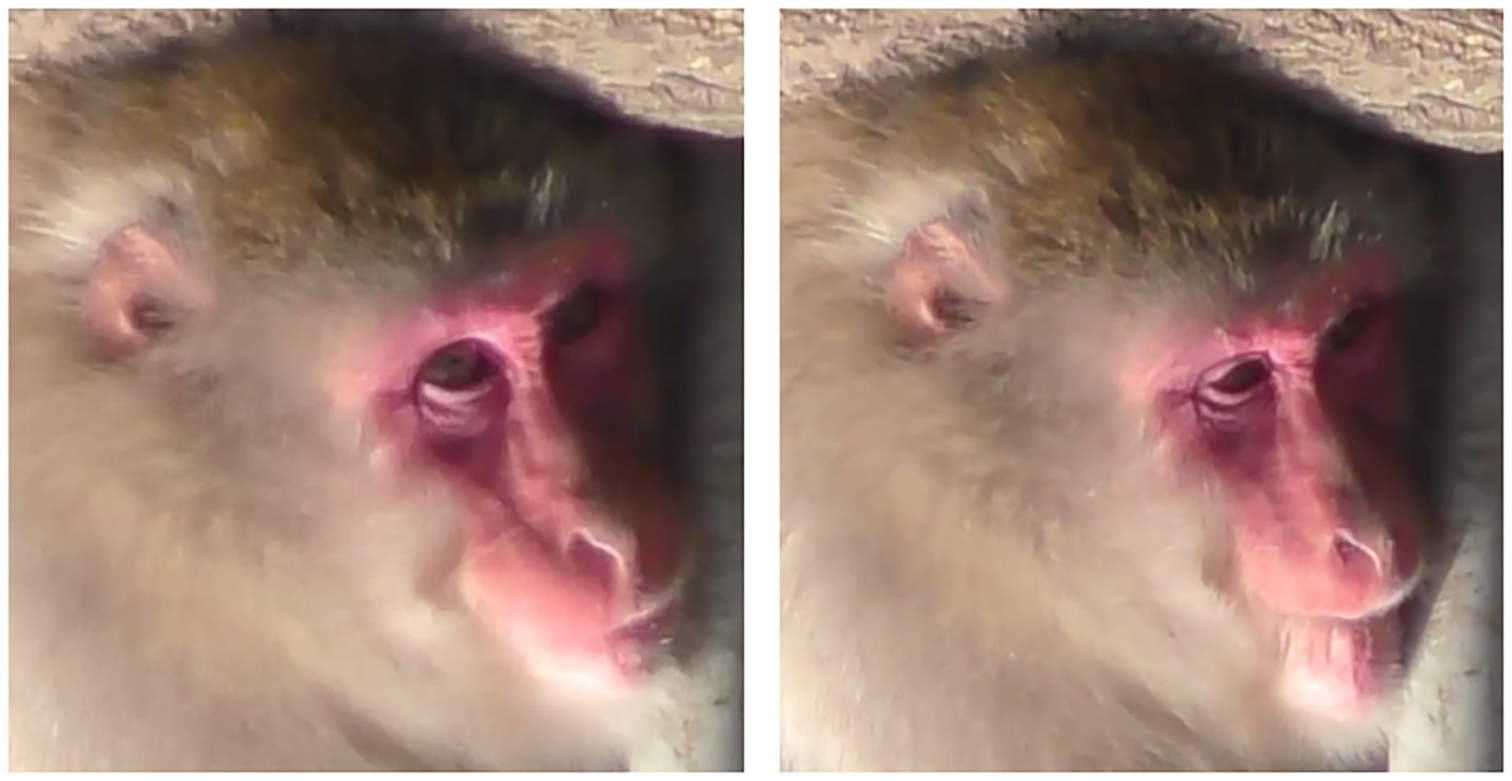
Left: Neutral lower face; Right: AU8 + AU18 +AU26, acting simultaneously with appearance changes from both AUs, namely stretched lower lip (AU8) and wrinkling of lower lip (AU18).

1) Comparative muscular basis: This orbicularis oris m. movement is described in humans as the vertical movement of the lips, with the upper lip being pulled towards the lower lip, and/or the lower lip pulled towards the upper lip, after mouth/jaw opening (AU26 or AU27), and without any inwards curvature of the lips.

3) Differences: While in humans, AU8 can happen with narrowing of the lips and together with AU18—Lip pucker (see below), in rhesus macaques AU18 and AU8 are described as mutually exclusive. However, in Japanese macaques, we observed AU8 on its own (S12a and S12b Video), but also appearance changes for AU8 and AU18 simultaneously, i.e. lips stretching vertically from AU8, and lip corners brought medially with wrinkles from AU18, S13a and S13b Video, Fig 6. Therefore, we suggest here a coding following the human FACS, allowing the coding of AU8+AU18 for Japanese macaques. Finally, AU8 can be confused with the closing of the mouth (release of AU25/AU26/AU27 by the masticatory muscles), but in AU8 the lips stretch and reach beyond their neutral size/position, giving an appearance of a cranio-caudally longer mouth.

#### AU9—Nose wrinkler and AU10—Upper lip raiser

1) Comparative muscular basis: In humans, AU9 and AU10 are observed clearly independently, with distinctive appearance changes produced by distinct muscles (levator labii superioris alaeque nasi and levator labii superioris, respectively). Despite the same independent muscles being identified in rhesus macaques, AU9 has not been observed without AU10, and therefore AU9+10 is usually coded.

2) Main appearance changes: Nonetheless, appearance changes for AU9 and AU10 are presented separately in the MaqFACS, and so AU10 can still be coded on its own. In Japanese macaques, some of the appearance changes are similar to rhesus macaques, but we found some differences as well (see below). Importantly, we observed AU9 and AU10 on its own a few times in different individuals, although AU9+10 was more often observed. Both AU9 and AU10 were observed unilaterally frequently (see examples below), but they can happen bilaterally as well.

#### AU9—Nose wrinkler

**Fig 7:**
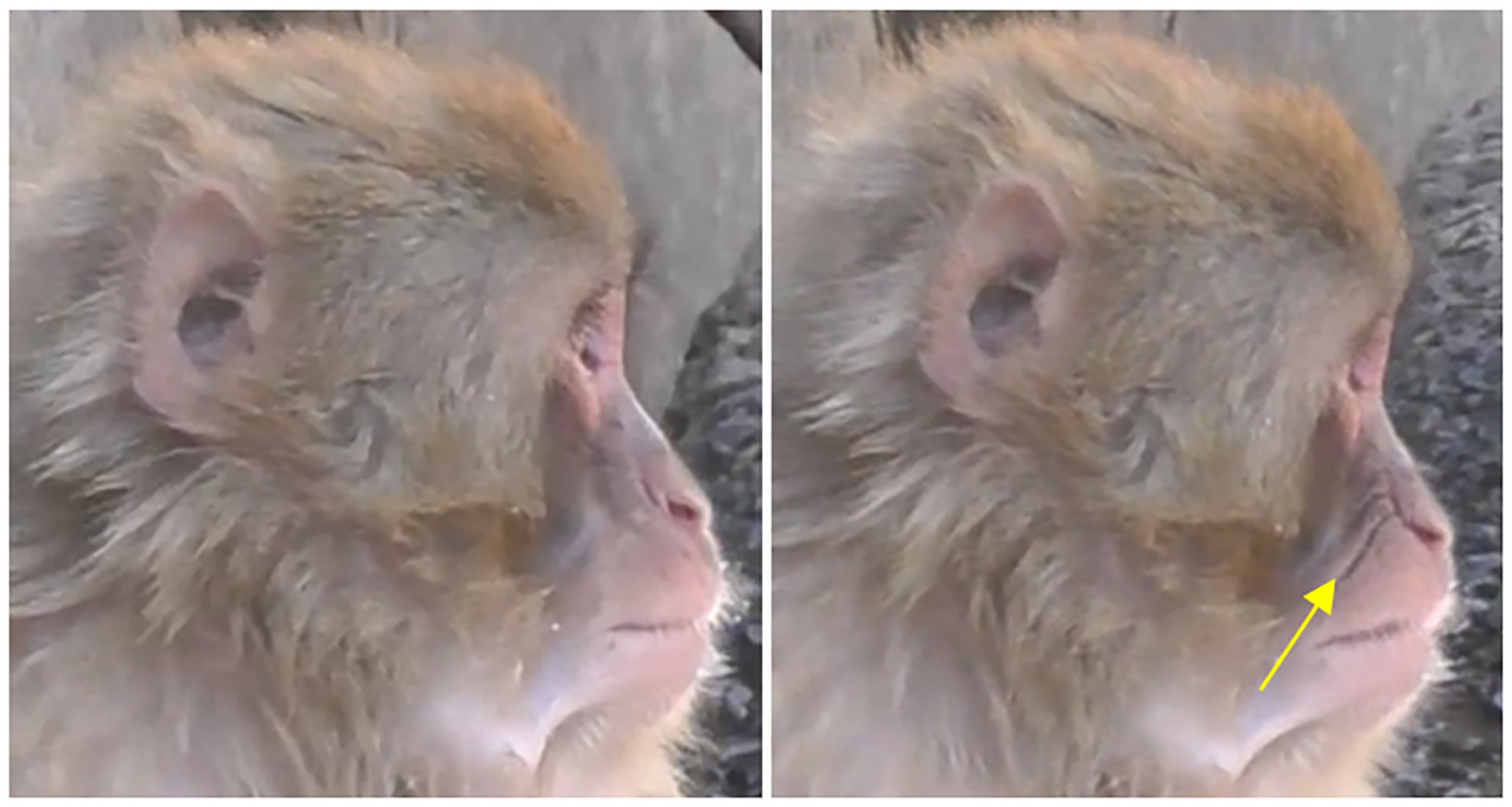
Left: Neutral; Right: AU9 (arrow indicates wrinkles oblique to the nose and shortening of IOT).

2) Main appearance changes: During AU9 (S14a and S14b Video), the main appearance change observed was an upwards movement towards the inner eye corner of the skin parallel to the nose creating oblique wrinkles along the nose on the IOT (Fig 7). The infraorbital triangle is shortened as well. Upwards movement on the nose and nostrils could also be seen during AU9, shortening the length of the nose and creating wrinkles on the nasal grove. The upper lip may be pulled upwards if AU9 is very strong, but no change in shape or size of the upper lip is observed.

#### AU10—Upper lip raiser

**Fig 8:**
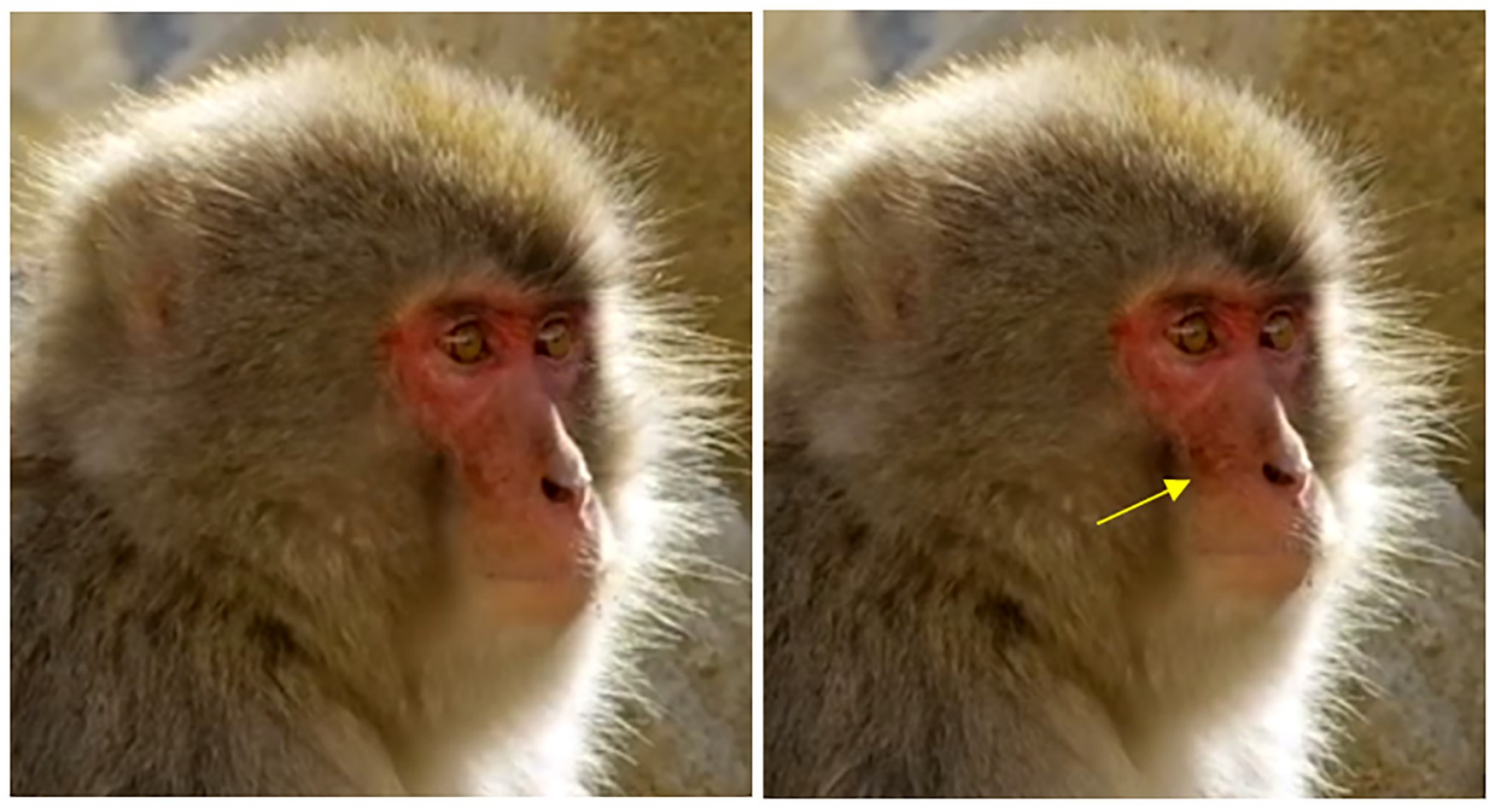
Left: Neutral; Right: AU10 with low intensity (arrow indicates wrinkles perpendicular to the nose and shortening of IOT).

2) Main appearance changes: In Japanese macaques, low or medium intensity AU10 was often raising the upper lip unilaterally (S15a and S15b Video, Fig 8) or within just the medial portion (S16a and S16b Video). We also observed bilateral stronger AU10 (S17a and S17b Video). This movement presents as either portions of the upper lip being pulled upwards or the whole upper lip being raised. In either case, shortening of the lip length vertically (and of the IOT) in the area of the movement is seen and wrinkles form immediately above the lip. The movement of the lip is more oblique than in 9+10, with wrinkles more perpendicular to the nose right above the lip (Fig 8). The shape of the upper lip may change, including the curvature of the lip and bulging may occur. Importantly, no movement is seen on the nose grove or nostrils, otherwise consider coding AU9+10.

#### AU9+10—Nose wrinkler + upper lip raiser

**Fig 9:**
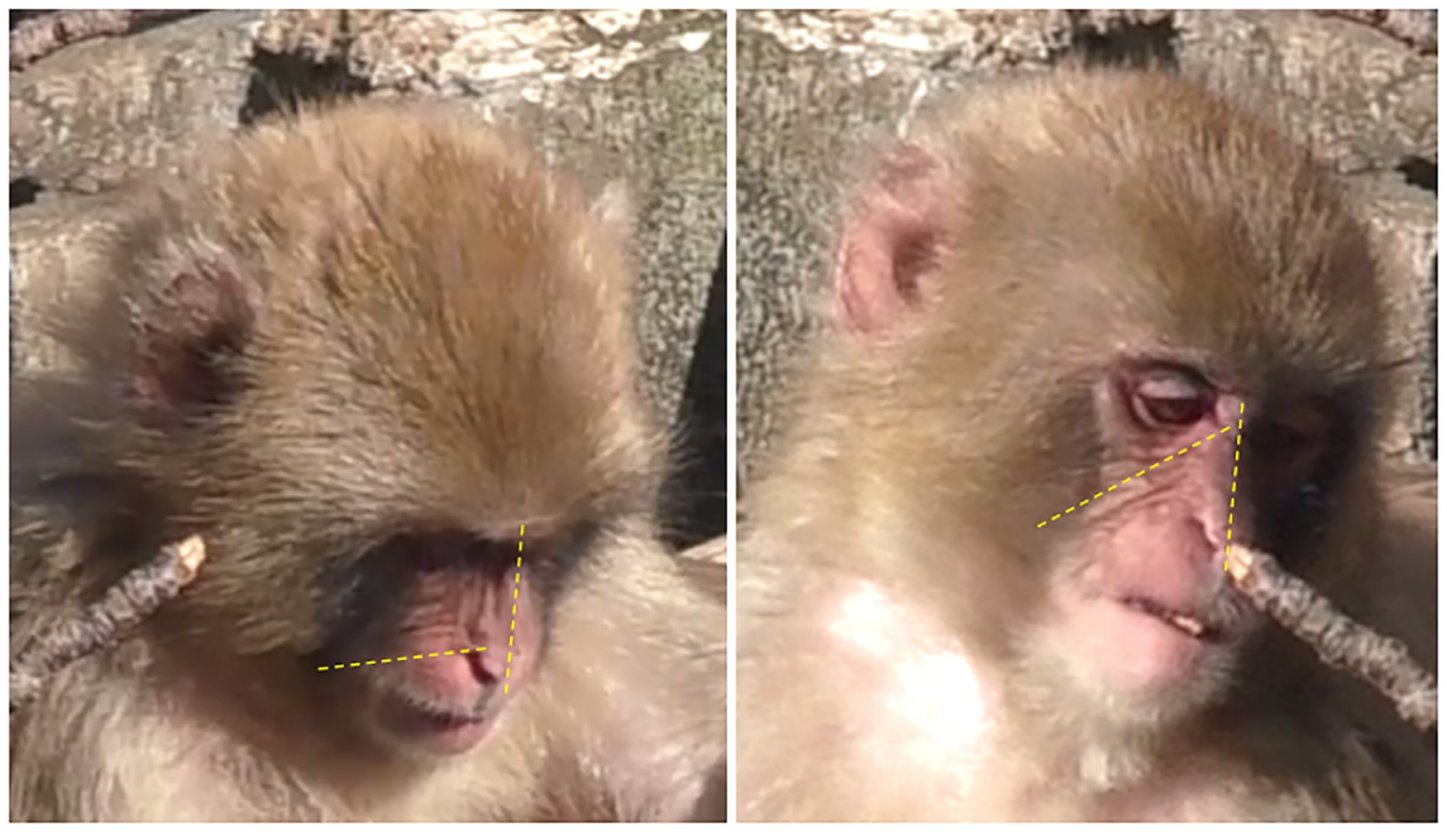
**Left: AU10 with perpendicular wrinkles to the nose**. Right: AU9+10 with wrinkles oblique to the nose (angle of wrinkles indicated by yellow dashed lines).

2) Main appearance changes: The main differences between AU9 and AU10 are the position of wrinkles and direction of pulling movement. While in AU9 the oblique wrinkles appear on the infraorbital triangle and sometimes extend to the nose, with a pulling movement towards the inner eye corner, in AU10 the wrinkles are almost perpendicular to the nose and appear on the lower edge of the infraorbital triangle, with a pulling movement more towards the outer corner of the eye (Fig 9). If appearance changes are not enough to code AU9 or AU10 independently, then code AU9+AU10 (S18a, S18b, S19a and S19b Videos), which seems to be the more frequent movement produced by Japanese macaques.

#### AU38—Nostril dilator

1) Comparative muscular basis: This movement was not described in the MaqFACS nor for Barbary macaques, but the nasalis m. movement was validated in rhesus macaques before (Waller et al 2008).

2) Main appearance changes: Here, we found an identical movement to what is seen in Waller et al (2008), with both nostrils being pulled outwards, appearing inflated (S20a and S20b Video).

#### AU12—Lip corner puller

1) Comparative muscular basis: In humans, the zygomaticus major m. pulls the corners of the lips back and upwards towards the ears, creating an upturned half-circle shape to the mouth. In rhesus macaques, the zygomaticus major m. is interrupted by the depressor anguli oris m., which is related to the opposite facial movement (i.e., lip corner depressor; Burrows et al. 2009; Parr et al. 2010).

2) Main appearance changes: Furthermore, in a neutral state, the lip corners of macaques are straight or slightly downwards. Thus, when AU12 acts, no upwards movements are seen. This anatomical feature was suggested to be shared by Japanese macaques [69]. And indeed, in Japanese macaques, we observed similar appearance changes (S21a, S21b, S22a and S22b Videos) to rhesus macaques. AU12 has been described before in neonatal Japanese macaques [69] as a feature of sleep. We also observed quick AU12 in adults while sleeping/dozing (S23a and S23b Video), which might indicate that this behaviour is not a feature of sleep in infants only, and thus quantification of AU12 in sleeping adults is needed in future studies for further comparisons.

#### AU16—Lower lip depressor

**Fig 10:**
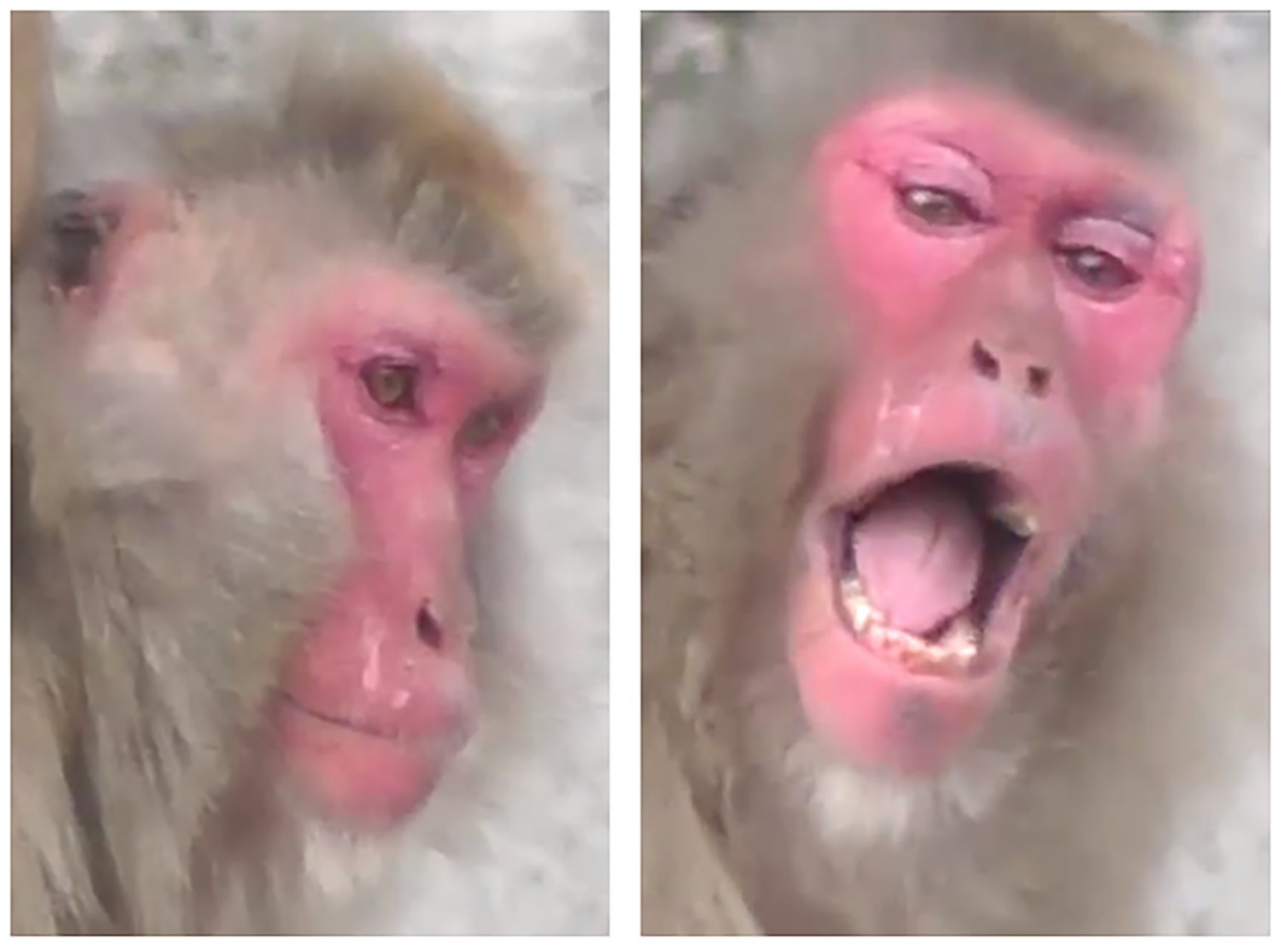
Left: Neutral; Right: AU16 with lower teeth exposure and orbital action moving the lower lip away from the teeth.

1) Comparative muscular basis: In humans and macaques, this movement is caused by the depressor labii inferioris m.

2) Main appearance changes: In the MaqFACS, AU16 is identified mainly by a change in the curvature of the lower lip, accompanied by a decrease in the distance between lower lip and mental region edge, and increase in lower teeth exposure. These appearance changes are also found in Japanese macaques (S24a and S24b Video).

3) Differences: However, in Japanese macaques we also observed some frequent orbital action added, with the lower lip being pushed away from the teeth protruding forward, forming a "V" shape in frontal view, like if it was inflated (Fig 10, S25a and S25b Video). In humans, AU16 has more marked appearance changes due to the chin. The chin is a unique anatomical feature in humans impacting appearance changes for AU16, but because macaques lack a chin bone and boss, appearance changes are more subtle.

#### AU17—Lower lip raiser (Chin raiser)

**Fig 11:**
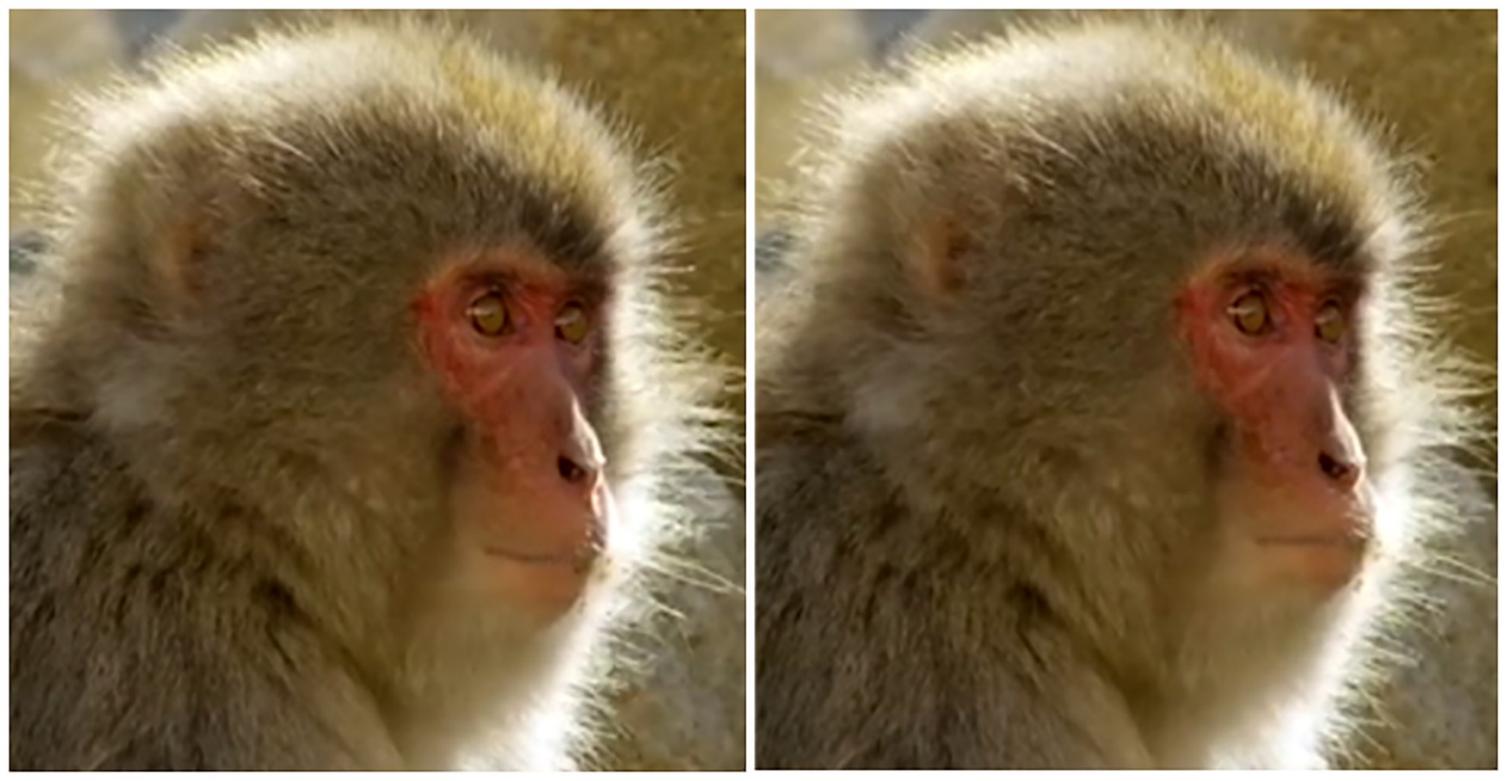
Left: Neutral; Right: AU17, slightly pushing the upper lip.

1) Comparative muscular basis: In humans, the chin raiser is produced by the action of the mentalis m. that pushes the lower lip upwards, protrudes the chin boss and wrinkles the chin. The bony chin boss is an anatomical feature unique to humans and its function is still highly debated [70]. Therefore, even though the muscular basis is the same, AU17 in other species is sometimes designated as Lower lip raiser (e.g. [19]).

2) Main appearance changes: The lack of chin boss impacts the appearance changes of AU17 in macaques, making it harder to detect. In the MaqFACS, it was only observed with other movements, but we observed it in isolation in Japanese macaques (S26a and S26b Video, Fig 11). The appearance changes for the Japanese macaques include: the lower lip is pushed upwards, the mental region skin is stretched, and the apparent size of the lower lip is increased. The upper lip might be pushed upwards by the lower lip if the mouth is closed and bulge, but the philtral region remains visible. If the philtral region is smoothed, AU24 might be acting as well (see below).

#### AU18i—True pucker and AU18ii—Outer pucker

**Fig 12:**
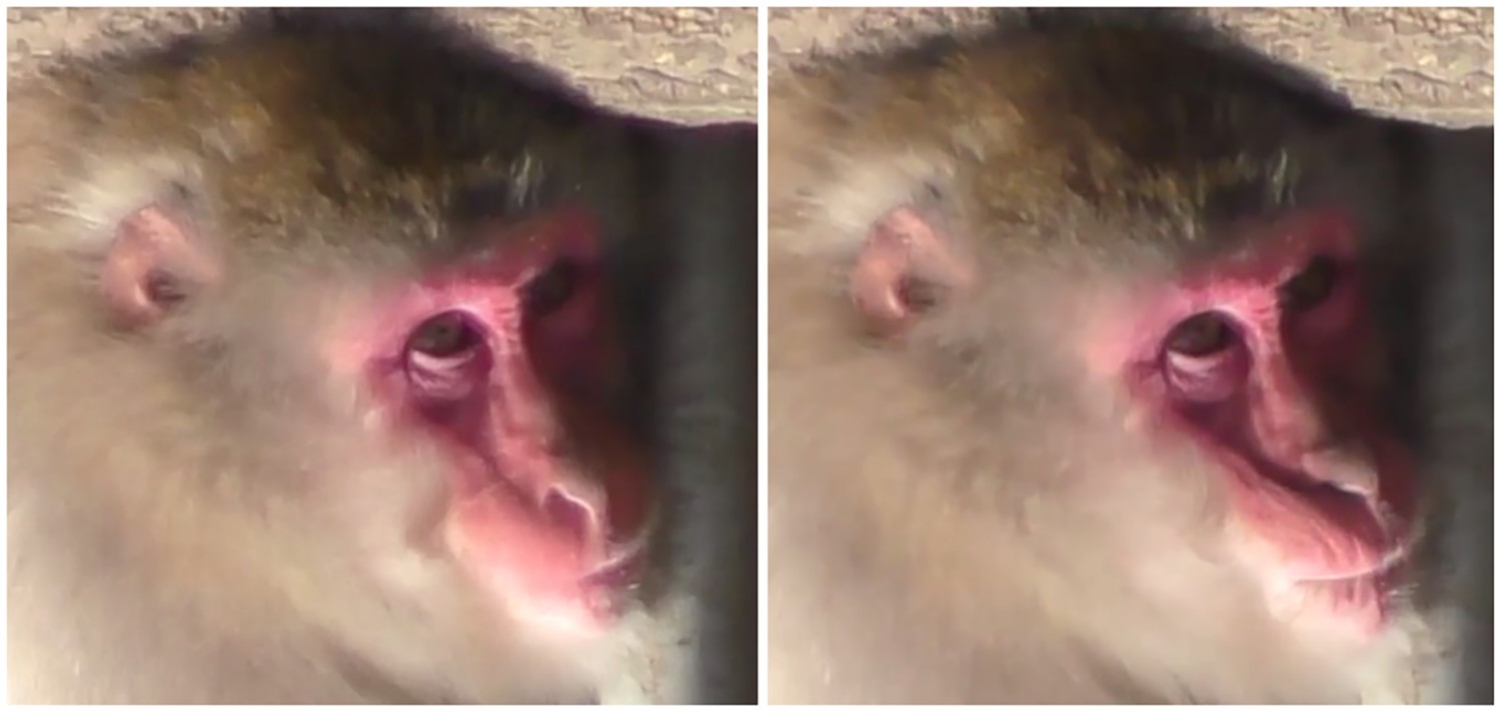
Left: Neutral; Right: AU18i.

**Fig 13:**
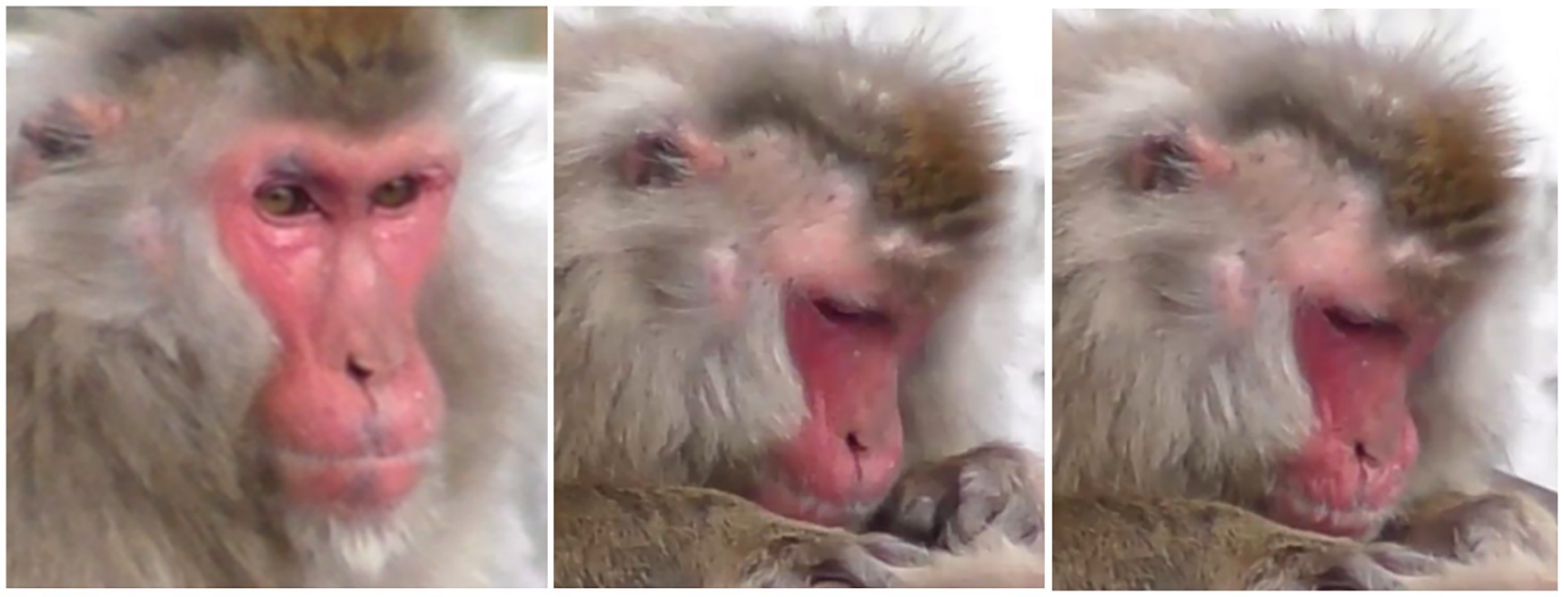
Left: Neutral; Right: AU18i during grooming.

**Fig 14:**
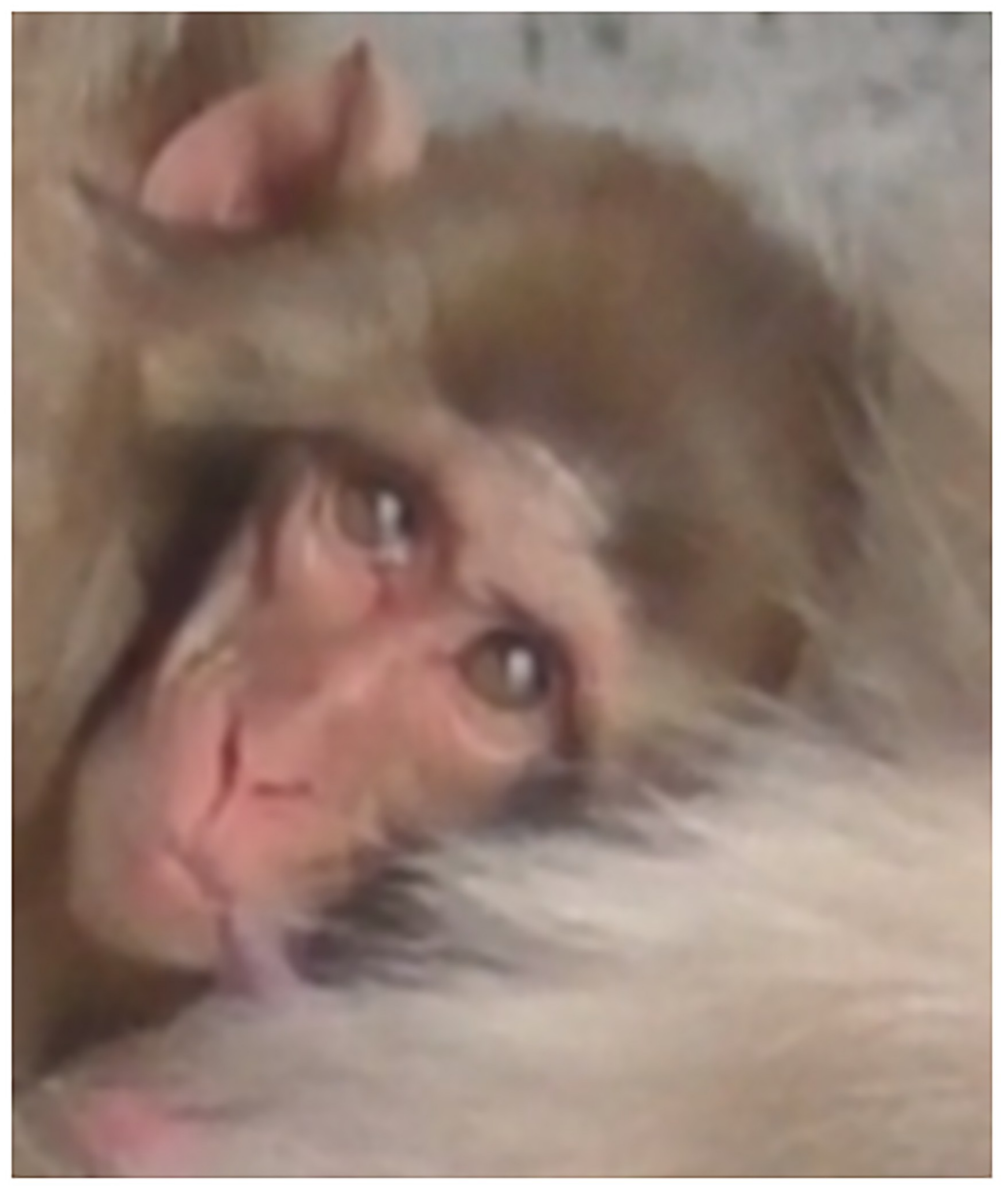
AU18i during nursing.

**Fig 15:**
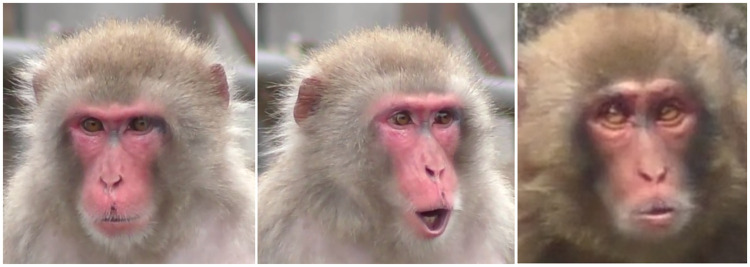
Left: Neutral; Centre: AU18i during vocalization (+AU16); Right: AU18ii.

1) Comparative muscular basis: In humans, AU18 brings the lip corners medially, creating wrinkles and protrusion of the everted lips. In rhesus macaques, due to differences in the protrusion of the lips and action of the orbicularis oris and incisivii labii muscles, two movements were described AU18i - True Pucker, equivalent to the human AU18, and AU18ii—Outer Pucker.

2) Main appearance changes: Similarly to rhesus macaques, Japanese macaques produce both AU18i (S27a and S27b Video, Figs 12–14) and AU18ii (S28 Video, Fig 15 Right).

3) Differences: For AU18i in Japanese macaques, we observed one difference to rhesus macaques: AU18i was both observed with and without protrusion/inflation of the lips. When there was no protrusion/inflation of the lips, the appearance changes were: 1) lip corners drawn medially, and/or 2) vertical wrinkles formed on both/either lips (Figs 12–14); therefore, in Japanese macaques these two appearance changes are enough to code AU18i. This AU18i without inflation or protrusion of the lips was observed often during not only vocalisations (Fig 15 **Centre**) and grooming (Fig 13), but also in nursing infants (Fig 14).

#### AU24—Lip presser

**Fig 16:**
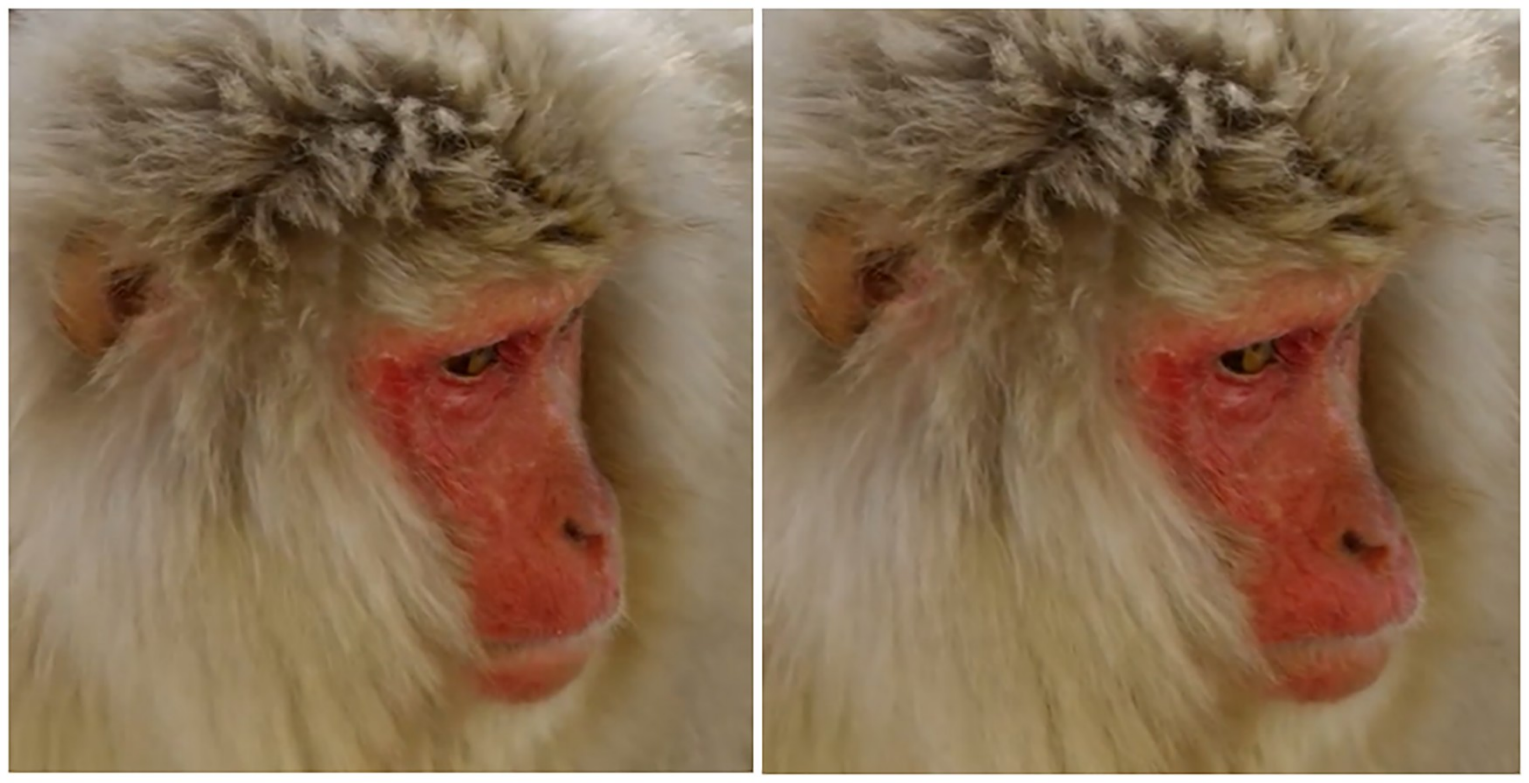
Left: Neutral; Right: AU24.

1) Comparative muscular basis: In humans, the orbicularis oris m. pushes the upper and lower lip against each other when the mouth is closed, creating wrinkles.

2) Main appearance changes: This movement was not described for rhesus or Barbary macaques, but it was observed for Japanese macaques (Fig 16, S29a and S29b Video). Here, the lips bulge, and appear narrower and tightened as they are pressed against each other, i.e. the distance between nose and upper lip, and lower lip to mental region edge decrease. The philtral region is less distinctive and smoother, the nose appears flattened against the face and the nostrils may decrease in size. Wrinkles may appear during this movement, but check the movement of the lip corners to see if the wrinkles are due to AU18 or AU24. If the lip corners are drawn medially during Lip Presser, then code AU18+AU24. Unlike AU18, this movement requires action of both lips, so only code AU24 if there is no AU25 and both lips are involved.

#### AU28—Lips suck and AD32—Bite

1) Comparative muscular basis: Due to the eversion and contrasting coloration of the lips in humans, AU28 is easier to identify, where in the main appearance change, the lip(s) are sucked or introduced into the mouth by the orbicularis oris m., after mouth opening (AU26).

2) Main appearance changes: This movement is not mentioned in the MaqFACS, but we observed it for Japanese macaques. Thus, the lips are introduced into the mouth folding around the teeth, stretching the skin above and below the lips (S30a and S30b Video). It can be observed in one lip only or lateralized as well (i.e. T28, B28, L28, R28). We also observed this movement followed by AD32—Lip Bite (S30a and S30b Video), where the individual then closes the jaw and bites the lip(s) holding them inside the mouth. The distinction between AU28 and AD32 depends on the orbicularis oris m. action: if the lip is placed inside the mouth by the orbicularis oris m. without any teeth aid, code AU28; if the teeth are pulling the lip and holding it inside the mouth, code AU32. Depending on the situation, the appearance changes may overlap or may be sequential. If clear distinction cannot be made, the human FACS advises coding AD32 only.

### Ear Action Units

#### EAU1—Ears forward, EAU2—Ears elevator, and EAU3—Ears flattener

1) Comparative muscular basis: Humans and macaques have three identical extrinsic ear muscles (anterior, superior and posterior auricularis m.). The ear movements in humans are very limited or non-existent [71], while in macaques these are an important part of the behavioural repertoire [65].

2) Main appearance changes: The ear movements observed in Japanese macaques were the same as in rhesus macaques, EAU1—Ears forward, EAU2—Ears elevator, and EAU3—Ears flattener (see MaqFACS for list of appearance changes).

3) Differences: Ear visibility is more limited in Japanese macaques in frontal view, which might also vary depending on the length and density of the individual’s hair. The ears might be more visible from a 3/4, side or back view, and even when the hair is covering the ears, EAUs can be ascertained by movement of the hair attached to the pinna, which in some individuals is of a lighter coloration than the rest of the head hair. Movement of the hair surrounding the ears is part of the appearance changes to code ear movements in rhesus macaques, and is even more important in Japanese macaques (S31 Video, where the ear is completely hidden, but the hair moves due to release of EAU3 and then activation of an EAU3). It is important to note that ear movements seem often coupled with head movement that may mask ear movement, so close attention to the position of the ear before and after head movements will aid in coding EAUs (e.g. EAU1 + AD51—Head turn left in S32a and S32b Video).

### Action descriptors

We observed several ADs (broader movements or movements from non-mimetic muscles) in Japanese macaques (Table 3). We briefly describe here these ADs and provide examples, as the co-occurrence of AUs and ADs can alter the appearance of the former, and some AUs seem to be often linked with some ADs (e.g. AU18 in Lipsmacking).

**Table 3:** Comparison between the Action Descriptors (ADs) previously included in the FACS developed for humans [67], rhesus [14] and Barbary [15] macaques, to what was here identified for Japanese macaques.

AD code	AD name	Human	Rhesus macaque^1^	Barbary macaque^1^	Japanese macaque
**AD101**	**Scalp Retraction**	**x**	**x**	**x**	✓
**AD181**	**Lip Smacking**	**x**	✓	✓	✓
			+AU18i	+bared-teeth display	
**AD19**	**Tongue Show**	✓	**x**	**x**	✓
**AD119**	**Lick**	✓	**x**	**x**	✓
**AD29**	**Jaw Thrust**	✓	✓	**x**	✓
**AD30**	**Jaw Sideways**	✓	✓	**x**	✓
**AD31**	**Jaw Clencher**	✓	**x**	**x**	**x**
**AD32**	**Bite**	✓	**x**	**x**	✓
**AD33**	**Blow**	✓	**x**	**x**	✓
**AD34**	**Puff**	✓	**x**	**x**	✓
**AD35**	**Suck**	✓	**x**	**x**	✓
**AD36**	**Bulge**	✓	**x**	**x**	✓
**AD37**	**Lip Wipe**	✓	**x**	**x**	**x**
**AD40**	**Sniff**	✓	**x**	**x**	**x**
**AD50**	**Vocalizations**	✓	**x**	**x**	✓
**AD80**	**Swallow**	✓	**x**	**x**	✓
**AD81**	**Chewing**	✓	**x**	**x**	✓
**AD86**	**Cheek Pouch Compressor**^2^	**x**	**x**	**x**	✓
**AD160**	**Body Shake**	**x**	**x**	**x**	✓

^1^Although these ADs are not described for rhesus or Barbary macaques, it is likely/possible some of these are present (e.g. Sniff) but have not been described by the authors.

^2^Cheek pouches are surrounded by the platysma (Burrows et al 2009, Burrows et al 2016).

#### AD101—Scalp retraction

1) Comparative muscular basis: The occipitalis m., one of the scalp muscles, is located between the galea aponeurotica (connective tissue covering top of the cranium) and the nuchal region, although in humans, it presents some variation between individuals regarding its length and insertion [72]. Functionally, it can accompany the contraction of another scalp muscle, the frontalis (during brow raiser), or the zygomaticus major (during lip corner puller), but it is generally activated independently, drawing back the galea aponeurotica only [73, 74] and moving the posterior part of the scalp superiorly [63]. Posterior scalp movements have not been reported as significant in human communication or emotional expression, although the occipitalis has been reported to be active during stressful tasks [75]. In macaques, the occipitalis m. is well‐defined and fully independent from all surrounding muscles, functionally similar to the human muscle, pulling the scalp posteriorly [61, 63, 76]. However, in *M*. *fuscata* (but not in *M*. *mulatta*), the occipitalis m. can be added to the frontalis action [4] to pull the scalp after lifting the browridge.

2) Main appearance changes: In Japanese macaques, we observed AD101—Scalp retraction frequently, but not independently from ear (EAU3) or brow (AU1+2) movements. Individuals might not be able or have difficulty in using these muscles independently in naturalistic behaviour (similar to what is seen in the human glabella muscles [68]) or it might not be part of their behavioural repertoire. It is also possible our footage did not capture this AD101 on its own due to perhaps being displayed in low intensity or particular contexts. Seiler [4] observed that *M*. *fuscata* pulled back the ears before lifting the browridge and ears strongly, which indicates that at least in high intensities, these movements might tend to act simultaneously. However, we did observe independent movements of brow raising (AU1+2) and ears (EAU3), and so the muscular activity reported in Seiler [4] might not necessarily translate as observable movement, as tensing a muscle might not produce visible movement of the skin or facial features. The appearance changes we observed in Japanese macaques during AD101 included posterior movement of the skin of the head, hair movement towards the nuchal region and subsequent flattening against the head, with AU1+2 and/or EAU3 acting simultaneously. When both these movements are added to AD101, the whole facial hair, including lateral facial crest, is pulled backwards and flattened, making the facial skin stretched and more exposed, with the face appearing larger (S33 and S34 Videos). It is possible that muscles such as the platysma are causing this more global head movement, likely causing the facial crest hair flattening and the stretching of the facial skin, similar to what happens in the lower face and neck of humans when the platysma is contracted (leading to AU21—Neck Tightener [67]).

3) Differences: Scalp retraction was not described in the MaqFACS nor in other detailed studies of rhesus macaques’ facial expressions (e.g. [77]). However, it has been included as a frequent behavioural category in ethograms of other macaque species (e.g. *M*. *nigra*: [78] and *M*. *tonkeana*: [79]) with very conspicuous appearance changes, such as posterior movement of the skin of the head, wrinkle formation on the occipital region, movement of the crest hair, ears flattening, and possibly brow movement. Very recently, scalp retraction has been included in a FACS adaptation for *M*. *nigra* with the code AD101, with the following description: "The hair on the top of the head, including the crest, flattens as the skin is pulled backward. Skin on the forehead and temples appears stretched.’ [16]. Despite the muscular basis being well studied in *M*. *fuscata*, we here apply this code for Japanese macaques as well since the movement does not appear to be an independent action (i.e. likely not an AU for *M*. *fuscata*).

Several macaque species display **Lipsmacking—AD181** in varied contexts [16, 77, 78, 80], and with variable presentation, where visual and auditory cues are combined. For example, in rhesus macaques [14], main appearance changes included lips being rapidly pressed together and relaxed. Lip curling, smack sounds, and a strong association with True pucker (AU18i) were also present. Since it was unclear if this succession of movements included a Lip presser (AU24) and/or a Lip tightener (AU23 [67]), it was instead combined into AD181. Another study [81] distinguished between open and closed-mouth lipsmacking in order to measure the rhythmic frequency of movement. In Barbary macaques [15], lipsmacking was described as having the same main appearance changes as in rhesus macaques, but with the addition of a bared-teeth/teeth chattering display, where the teeth are visible and accompany the lip movement, producing a sound. For the crested macaque [16], additional ADs for teeth chattering (AD182) and tongue chattering (AD183) help distinguishing these from lipsmacking (AD181). In Japanese macaques, we observed lipsmacking with and without AU18 and AU25, but further comprehensive studies on the combination and presentation of these movements and sounds are needed to fully characterise lipsmacking in macaques.

**AD19—Tongue Show**, was often seen in Japanese macaques, either on its own or accompanying other movements. This movement is coded whenever the tongue is shown beyond the teeth, where the mouth is open (AU26) and the lips parted (AU25). Another movement we observed with the tongue was **AD36—Bulge**, where the tongue is pushed against the cheek or lip, causing the skin to stretch on that area and to bulge.

Two jaw movements described for humans were also observed in Japanese macaques: **AD29—Jaw Thrust** and **AD30—Jaw Sideways**, where AD29 codes a forward displacement of the jaw protruding the mental region and lower lip, while AD30 codes the displacement of the jaw to one side of the face, creating a misaligned lower face with the upper face.

The expansion or deflation of the cheeks are described in human FACS as **AD34—Puff and AD35—Suck**, and identical movements were found in Japanese macaques, with AD34 expanding the cheek skin as air is forced into the mouth with the lips closed, and AD35 sucks the cheeks into the mouth producing a depression anterior and caudally to the lip corners. We also observed individuals compressing the cheek pouches, raising the skin of the cheek and neck area by the contraction of the platysma m. [61, 63], and pushing the lip corners and upper lip cranially and medially, which may impact lower face AUs (e.g. AU18). While compressing the cheeks, the lip corners may be pushed forward, but there are no wrinkles on the lips; the upper lip may be pushed cranially, but the movement is seen from coming from the neck/cheek area, instead of the IOT. Hence, we created the **AD86—Cheek Pouch Compressor** to code this movement. We observed movement of food from the cheek pouches to the cheeks and then disappearing caudally (i.e. by being swollen), which sometimes was accompanied by facial movements, such as AU18 (S35 and S36 Videos). Japanese macaques would sometimes push food with their hands from the pouches to the mouth, together with AD86.

**Fig 17:**
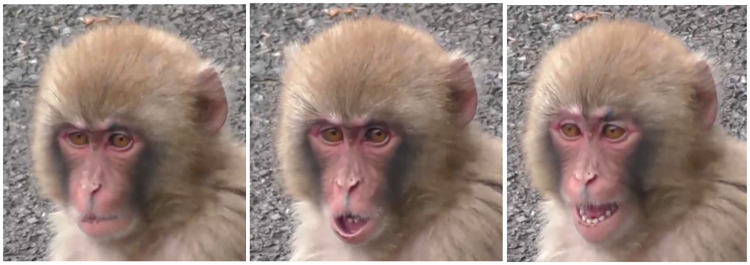
Left: Neutral; Centre and Right: varied AUs during AD50—Vocalization.

Finally, we mention here **AD50—Vocalizations**, as Japanese macaques were observed to produce varied facial movements during vocalizations, modifying slightly the appearance changes of the corresponding AUs. For example, AU18ii was observed together with AU16 in the lower lip, pushing the lip away from the teeth, which was not observed without the vocalization (e.g. Fig 17, S37 Video).

In total, we identified 19 AUs, 16 ADs and 3 EAUs in Japanese macaques (Tables 2 and 3), which indicates a similar or slightly higher facial mobility than what was described for rhesus and Barbary macaques (15 AUs, 1–3 ADs, 3 EAUs), but lower than in humans (30 AUs, 25 ADs, no EAU). Although the ADs described here for Japanese macaques is considerably higher than for rhesus or Barbary macaques, it is possible that some of the AUs described for Japanese macaques are also present in the other species (e.g. Sniff) but have not been described by the authors.

## General discussion

Our current work aimed at developing a new FACS tool to objectively measure facial expressions in Japanese macaques, following FACS methodology previously used since the original HumanFACS [67] to the latest FACS published for non-human species [19]. In the first step of investigating the muscular anatomical plan of Japanese macaques, the dissection-literature available indicated that rhesus and Japanese macaques have identical facial musculature. Since rhesus macaques had a FACS developed already (MaqFACS [14]), and each new FACS is rooted on the anatomical plan of the species, it rendered it unnecessary to create a new FACS for Japanese macaques. However, morphologically, rhesus and Japanese macaques are still considerably different, and so we still needed to check the application of MaqFACS to Japanese macaques, note differences between what was reported in MaqFACS and what we observed in Japanese macaques, and finally add new movements if observed. All movements from the MaqFACS were observed and we were able to code all of them in Japanese macaques, with differences in appearance changes and morphology noted, as well as some new movements documented, not present in the MaqFACS. Hence, our work here resulted in the *MaqFACS Extension for Japanese macaques* (similar to the work developed before for Barbary macaques [15]), which is recommended to be used as an additional resource to the MaqFACS manual for rhesus macaques. Only if coders are certified in MaqFACS and use its MaqFACS Extension when coding Japanese macaques, can the robustness of the system be maintained.

The number of AUs described for Japanese macaques was similar to what was described for other macaques (rhesus and Barbary) as well as other primates (chimpanzees: 15 AUs, orangutans: 17 AUs and gibbons: 20 AUs), suggesting that Japanese macaques, as other primate species, have a wide range of facial movements that can potentially be used to compose a variety of facial expressions. However, it is important to note that our study did not measure the actual use or co-occurrence of these movements in this species, but by applying the *MaqFACS Extension for Japanese macaques*, future research can now objectively measure this species’ facial movements.

For example, in future studies accounting for the socio-ecological factors of Japanese macaques and other macaque species, it might be interesting to look at the relationship between facial movement complexity (i.e. with more AUs/higher co-occurrence of AUs) and social styles (i.e. despotic-tolerant), as well as other factors (e.g. group size, habitat variety) in order to understand how evolutionary pressures might have shaped communication in each species. All mammals have well conserved facial musculature [82] and therefore, even though the potential for facial movement is similar across mammals, the use of these muscles varies from species to species [83]. Additionally, the sole presence of a particular muscle does not necessarily translate into: a) a functional independent movement (e.g. due to phylogenetic inertia), and/or b) the same appearance changes (e.g. due to facial morphological differences). This was observed during the current work, for example for the AU4/AU41—Brow/Glabella lowerer, where all macaques have the same muscles as in humans, but the observable movement is distinct (no corrugation is observed). In Japanese macaques, the isolation in Japanese islands, the variety of environment they adapted to, the large face/body (with low sexual dimorphism), and their large social groups might have diversified both the facial morphology and the facial movements in this species more than predicted by other factors.

Hence, although Darwin’s initial idea of emotional continuity between species [1] is fitting regarding the anatomical basis, it does not seem to completely explain facial expression diversity, particularly if we analyse the use of facial movements, since facial movements seem highly flexible and adaptative depending on the species. Interestingly, facial expressions might be classed as highly specialised behaviours, and thus, a product of evolutionary pressures from varied socio-ecological factors that set species apart. Even if movements are morphologically similar, their meaning will likely not be the same, resembling the extensive variation and isolation seen in human languages [84]. These outstanding questions further demonstrate the need for objective and detailed tools like MaqFACS to accurately and systematically measure facial movements in different species in order to understand how the facial movements translate into meaningful visual information.

Another explanation for this diversity of facial movements, might be that in Japanese macaques (as with probably other macaques), facial expressions are not exclusively used for communicative (i.e. intentionally *sensu* [85]) purposes, but also used in emotional (i.e. linked to neural primary systems *sensu* [86]) expression (notwithstanding that these are not necessarily mutually exclusive, but for more on this debate see [87]). Japanese macaques are the third most used macaque species for laboratory work, and one of the most used for painful biomedical experiments, due to their reputation as similar to humans in their neurobiology as well as cognition [56]. For example, in crab-eating macaques [54, 55] facial movements have been used as a marker of pain, therefore focus on such emotional expressions using FACS might reveal more about Japanese macaques’ diversity of facial behaviours as well as potentially aid in pain assessment in captive individuals. However, given the reduced literature on Japanese macaques’ facial expressions in general (i.e. the few studies published assume no differences from other species of *Macaca*), it is more likely that this species’ facial expressions are simply poorly studied, and so the *MaqFACS Extension* will be a valuable resource from now on.

Although this has not yet been measured with FACS in primates, the human FACS has been used to code asymmetric AUs that might potentially be associated with emotional states due to the lateralised brain processes (see [88] for a review). Asymmetry in animal facial expression has also been documented [89], but might be difficult to quantify without FACS. Both Japanese and Barbary macaques display asymmetrical AUs, with the most obvious one being AU1+2U that is coded when the browridge is raised more or only on one hemiface. Future research can elucidate how common these asymmetrical AUs are, when they occur or if they are indeed associated with emotional contexts.

Although we did not systematically quantify durations, we noticed that some facial movements in Japanese macaques were extremely quick and short (e.g. under 9 frames or 0.15 sec). This was noted before for other species (e.g. [13]), where slow motion clips are required to actually *see* the movements. This is especially interesting when compared with human facial movements, which under 200-500ms are considered micro-expressions and usually require training to be identified accurately [90, 91]. Human micro-expressions are also of particular interest as it has been suggested these are related to leakage of concealed emotions (Ekman 2003). On the other hand, the human visual conscious perception limit is between 20 to 40ms, depending on task and individual variation [92, 93]; Consequently, some (if not many) of the facial movements in non-human animals might need additional training for detection (if <500ms) or might not even be detectable at all by humans in real time (if <40ms). If this is the case, studies looking into macaques’ facial expressions likely require video recordings, with high-frame rate for slow motion options and more detailed analysis, as well as FACS training coders that are able to detect subtle facial motion.

Animal FACS have been used to investigate a range of both applied and fundamental questions, ranging from applying DogFACS to investigate emotion in dogs [83, 94] as well as to control the content of facial visual stimuli in cognition experiments [25], to applying OrangFACS to determine intentionality of communication in orangutans’ play [21]. With the *MaqFACS Extension for Japanese macaques*, it is now possible to objectively and systematically quantify a variety of facial behaviour variables (e.g. frequency, duration co-occurrence and lateralisation of each AU) in order to better understand Japanese macaques’ expressions as a species. Furthermore, it is a powerful comparative tool that will allow comparisons to other species using the same tools, in order to further explore the evolutionary pathways of communication and emotion processes in animals and humans.

## Supporting information

S1 Text (DOCX)

S1 Video **a**. Video example of AU1+2 in real time. **b**. Video example of AU1+2 in slow motion.
(ZIP)

S2 Video **a**. Video example of AU1+2 in real time. **b**. Video example of AU1+2 in slow motion.
(ZIP)

S3 Video **a**. Video example of AU1+2 in real time. **b**. Video example of AU1+2 in slow motion.
(ZIP)

S4 Video Video example of AU1+2 in real time from a top view. (AVI)

S5 Video **a**. Video example of unilateral AU1+2 in real time. **b**. Video example of unilateral AU1+2 in slow motion.
(ZIP)

S6 Video Video example of unilateral AU1+2 in real time. (MP4)

S7 Video **a**. Video example of AU41 in real time. **b**. Video example of AU41 in slow motion.
(ZIP)

S8 Video Video example of AU41 in real time. (AVI)

S9 Video Video example of AU41 in real time. (AVI)

S10 Video **a**. Video example of AU43 and AU6 in real time. **b**. Video example of AU43 and AU6 in slow motion.
(ZIP)

S11 Video **a**. Video example of AU6 during a yawn in real time. **b**. Video example of AU6 during a yawn in slow motion.
(ZIP)

S12 Video **a**. Video example of AU8 in real time. **b**. Video example of AU8 in slow motion.
(ZIP)

S13 Video **a**. Video example of AU8 and AU18 in real time. **b**. Video example of AU8 and AU18 in slow motion.
(ZIP)

S14 Video **a**. Video example of AU9 in real time. **b**. Video example of AU9 in slow motion.
(ZIP)

S15 Video **a**. Video example of AU10 in real time. **b**. Video example of AU10 in slow motion.
(ZIP)

S16 Video **a**. Video example of AU10 in real time. **b**. Video example of AU10 in slow motion.
(ZIP)

S17 Video **a**. Video example of AU10 in real time. **b**. Video example of AU10 in slow motion.
(ZIP)

S18 Video **a**. Video example of AU9+10 in real time. **b**. Video example of AU9+10 in slow motion.
(ZIP)

S19 Video **a**. Video example of AU9+10 in real time. **b**. Video example of AU9+10 in slow motion.
(ZIP)

S20 Video **a**. Video example of AU38 in real time. **b**. Video example of AU38 in slow motion.
(ZIP)

S21 Video **a**. Video example of AU12 in real time. **b**. Video example of AU12 in slow motion.
(ZIP)

S22 Video **a**. Video example of AU12 in real time. **b**. Video example of AU12 in slow motion.
(ZIP)

S23 Video **a** Video example of AU12 in real time. **b**. Video example of AU12 in slow motion.
(ZIP)

S24 Video **a**. Video example of AU16 in real time. **b**. Video example of AU16 in slow motion.
(ZIP)

S25 Video **a**. Video example of AU16 in real time. **b**. Video example of AU16 in slow motion.
(ZIP)

S26 Video **a**. Video example of AU17 in real time. **b**. Video example of AU17 in slow motion.
(ZIP)

S27 Video **a**. Video example of AU18i in real time. **b**. Video example of AU18i in slow motion.
(ZIP)

S28 Video Video example of AU18ii in real time. (AVI)

S29 Video **a**. Video example of AU24 in real time. **b**. Video example of AU24 in slow time.
(ZIP)

S30 Video **a**. Video example of AU28 followed by AD32 in real time. **b**. Video example of AU28 followed by AD32 in slow motion.
(ZIP)

S31 Video Video example of EAU3 release followed by EAU3 action in real time. (MP4)

S32 Video **a**. Video example of EAU1 and AD51 in real time. **b**. Video example of EAU1 and AD51 in slow motion.
(ZIP)

S33 Video **a**. Video example of AU100 during a yawn in real time. **b**. Video example of AU100 during a yawn in slow motion.
(ZIP)

S34 Video **a**. Video example of AU100 accompanied by AU1+2 and EAU3 from back view in real time. **b**. Video example of AU100 accompanied by AU1+2 and EAU3 from back view in slow motion.
(ZIP)

S35 Video **a**. Video example of AD86 in real time. **b**. Video example of AD86 in slow motion.
(ZIP)

S36 Video **a**. Video example of AD86 in real time. **b**. Video example of AD86 in slow motion.
(ZIP)

S37 Video Video example of AD50 accompanied by several other AUs in real time. (MP4)

## References

[1] Darwin C. The expression of the emotions in man and animals. New York: D. Appleton and Company; 1896.

[2] Ekman P, Friesen WV. Facial coding action system (FACS): A technique for the measurement of facial actions. Palo Alto, CA: Consulting Psychologists Press; 1978.

[3] Bruce V, Young AW. Face perception. New York: Psychology Press; 2012.

[4] Seiler R. On the function of facial muscles in different behavioral situations. A study based on muscle morphology and electromyography. American Journal of Physical Anthropology. 1973;38: 567–571. 10.1002/ajpa.1330380268 4632105

[5] Horowitz A, Hecht J. Looking at Dogs: Moving from Anthropocentrism to Canid Umwelt In: Horowitz A, editor. Domestic Dog Cognition and Behavior: The Scientific Study of Canis familiaris. Berlin, Heidelberg: Springer; 2014 pp. 201–219. 10.1007/978-3-642-53994-7_9

[6] Masataka N, Fujii H. An experimental study on facial expressions and interindividual distance in Japanese macaques. Primates. 1980;21: 340–349. 10.1007/BF02390464

[7] Maréchal L, Levy X, Meints K, Majolo B. Experience-based human perception of facial expressions in Barbary macaques (Macaca sylvanus). PeerJ. 2017;5: e3413 10.7717/peerj.3413 28584731 PMC5457665

[8] Hess U, Adams RB, Simard A, Stevenson MT, Kleck RE. Smiling and sad wrinkles: Age-related changes in the face and the perception of emotions and intentions. J Exp Soc Psychol. 2012;48: 1377–1380. 10.1016/j.jesp.2012.05.018 23144501 PMC3491992

[9] de Waal FBM, Luttrell LM. The formal hierarchy of rhesus macaques: An investigation of the bared-teeth display. American Journal of Primatology. 1985;9: 73–85. 10.1002/ajp.1350090202 32102494

[10] Ekman P, Friesen WV. Felt, false, and miserable smiles. J Nonverbal Behav. 1982;6: 238–252. 10.1007/BF00987191

[11] Waller BM, Julle-Daniere E, Micheletta J. Measuring the evolution of facial ‘expression’ using multi-species FACS. Neuroscience & Biobehavioral Reviews. 2020;113: 1–11. 10.1016/j.neubiorev.2020.02.031 32105704

[12] Parr LA, Waller BM, Vick SJ, Bard KA. Classifying chimpanzee facial expressions using muscle action. Emotion. 2007;7: 172–181. 10.1037/1528-3542.7.1.172 17352572 PMC2826116

[13] Caeiro CC, Waller BM, Zimmermann E, Burrows AM, Davila-Ross M. OrangFACS: A muscle-based facial movement coding system for orangutans (Pongo spp.). Int J Primatol. 2013;34: 115–129. 10.1007/s10764-012-9652-x

[14] Parr LA, Waller BM, Burrows AM, Gothard KM, Vick SJ. Brief communication: MaqFACS: A muscle-based facial movement coding system for the rhesus macaque. American Journal of Physical Anthropology. 2010;143: 625–630. 10.1002/ajpa.21401 20872742 PMC2988871

[15] Julle-Danière É, Micheletta J, Whitehouse J, Joly M, Gass C, Burrows AM, et al MaqFACS (Macaque Facial Action Coding System) can be used to document facial movements in Barbary macaques (Macaca sylvanus). PeerJ. 2015;3: e1248 10.7717/peerj.1248 26401458 PMC4579026

[16] Clark PR, Waller BM, Burrows AM, Julle‐Danière E, Agil M, Engelhardt A, et al Morphological variants of silent bared-teeth displays have different social interaction outcomes in crested macaques (Macaca nigra). American Journal of Physical Anthropology. 2020;173: 411–422. 10.1002/ajpa.24129 32820559

[17] Waller BM, Lembeck M, Kuchenbuch P, Burrows AM, Liebal K. GibbonFACS: A muscle-based facial movement coding system for hylobatids. International Journal of Primatology. 2012;33: 809–821. 10.1007/s10764-012-9611-6

[18] Waller BM, Peirce K, Caeiro CC, Scheider L, Burrows AM, McCune S, et al Paedomorphic facial expressions give dogs a selective advantage. PLOS ONE. 2013;8: e82686 10.1371/journal.pone.0082686 24386109 PMC3873274

[19] Caeiro CC, Burrows AM, Waller BM. Development and application of CatFACS: Are human cat adopters influenced by cat facial expressions? Applied Animal Behaviour Science. 2017;189: 66–78. 10.1016/j.applanim.2017.01.005

[20] Wathan J, Burrows AM, Waller BM, McComb K. EquiFACS: The Equine Facial Action Coding System. Hillmann E, editor. PLOS ONE. 2015;10: e0131738 10.1371/journal.pone.0131738 26244573 PMC4526551

[21] Waller BM, Caeiro CC, Davila-Ross M. Orangutans modify facial displays depending on recipient attention. PeerJ. 2015;3: e827 10.7717/peerj.827 25802802 PMC4369341

[22] Parr LA, Waller BM, Heintz M. Facial expression categorization by chimpanzees using standardized stimuli. Emotion. 2008;8: 216–231. 10.1037/1528-3542.8.2.216 18410196 PMC2826112

[23] Scheider L, Waller BM, Oña L, Burrows AM, Liebal K. Social Use of Facial Expressions in Hylobatids. Zimmermann E, editor. PLOS ONE. 2016;11: e0151733 10.1371/journal.pone.0151733 26978660 PMC4792372

[24] Hare B, Tomasello M. Human-like social skills in dogs? Trends in Cognitive Sciences. 2005;9: 439–444. 10.1016/j.tics.2005.07.003 16061417

[25] Correia-Caeiro C, Guo K, Mills DS. Perception of dynamic facial expressions of emotion between dogs and humans. Anim Cogn. 2020;23: 465–476. 10.1007/s10071-020-01348-5 32052285 PMC7181561

[26] Descovich K, Wathan JW, Leach MC, Buchanan-Smith HM, Flecknell P, Farningham D, et al Facial expression: An under-utilized tool for the assessment of welfare in mammals. ALTEX: Alternatives to Animal Experimentation. 2017;34: 409–429. 10.14573/altex.1607161 28214916

[27] Camerlink I, Coulange E, Farish M, Baxter EM, Turner SP. Facial expression as a potential measure of both intent and emotion. Scientific Reports. 2018;8: 17602 10.1038/s41598-018-35905-3 30514964 PMC6279763

[28] Gleerup KB, Andersen PH, Wathan J. What information might be in the facial expressions of ridden horses? Adaptation of behavioral research methodologies in a new field. Journal of Veterinary Behavior. 2018;23: 101–103. 10.1016/j.jveb.2017.12.002

[29] Tuyttens FAM, de Graaf S, Heerkens JLT, Jacobs L, Nalon E, Ott S, et al Observer bias in animal behaviour research: can we believe what we score, if we score what we believe? Animal Behaviour. 2014;90: 273–280. 10.1016/j.anbehav.2014.02.007

[30] Li J, Han K, Xing J, Kim H-S, Rogers J, Ryder OA, et al Phylogeny of the macaques (Cercopithecidae: Macaca) based on Alu elements. Gene. 2009;448: 242–249. 10.1016/j.gene.2009.05.013 19497354 PMC2783879

[31] Thierry B, Iwaniuk AN, Pellis SM. The Influence of Phylogeny on the Social Behaviour of Macaques (Primates: Cercopithecidae, genus Macaca). Ethology. 2000;106: 713–728. 10.1046/j.1439-0310.2000.00583.x

[32] Maestripieri D. Gestural communication in three species of macaques (Macaca mulatta, M. nemestrina, M. arctoides): Use of signals in relation to dominance and social context. Gesture. 2005;5: 57–73. 10.1075/gest.5.1.06mae

[33] Maestripieri D. Gestural Communication in Macaques: Usage and Meaning of Nonvocal Signals. Evolution of Communication. 1997;1: 193–222. 10.1075/eoc.1.2.03mae

[34] Ikeda J, Watanabe T. Morphological studies of Macaca fuscata. Primates. 1966;7: 271–288. 10.1007/BF01730793

[35] Richard AF, Goldstein SJ, Dewar RE. Weed macaques: The evolutionary implications of macaque feeding ecology. Int J Primatol. 1989;10: 569 10.1007/BF02739365

[36] Dobson M, Kawamura Y. Origin of the Japanese land mammal fauna: Allocation of extant species to historically-based categories. Quaternary Res. 1998;37: 385–395.

[37] Yamagiwa J, Hill DA. Intraspecific variation in the social organization of Japanese macaques: Past and present scope of field studies in natural habitats. Primates. 1998;39: 257–273. 10.1007/BF02573076

[38] Fooden J. Comparative Review of Fascicularis-group Species of Macaques (primates: Macaca). fzoo. 2006;2006: 1–43. 10.3158/0015-0754(2006)107[1:CROFSM]2.0.CO;2

[39] Thierry B, Aureli F, Nunn CL, Petit O, Abegg C, de Waal FBM. A comparative study of conflict resolution in macaques: insights into the nature of trait covariation. Animal Behaviour. 2008;75: 847–860. 10.1016/j.anbehav.2007.07.006

[40] Preuschoft S. Primate Faces and Facial Expressions. Social Research. 2000;67: 245–271.

[41] Dobson SD. Socioecological correlates of facial mobility in nonhuman anthropoids. American Journal of Physical Anthropology. 2009;139: 413–420. 10.1002/ajpa.21007 19235791

[42] Dobson SD. Coevolution of Facial Expression and Social Tolerance in Macaques. American Journal of Primatology. 2012;74: 229–235. 10.1002/ajp.21991 24006541

[43] Aureli F, Das M, Veenema HC. Differential kinship effect on reconciliation in three species of macaques (Macaca fascicularis, M. fuscata, and M. sylvanus). Journal of Comparative Psychology. 1997;111: 91–99. 10.1037/0735-7036.111.1.91 9090139

[44] Sueur C, Petit O, De Marco A, Jacobs AT, Watanabe K, Thierry B. A comparative network analysis of social style in macaques. Animal Behaviour. 2011;82: 845–852. 10.1016/j.anbehav.2011.07.020

[45] Dobson SD. Allometry of facial mobility in anthropoid primates: Implications for the evolution of facial expression. American Journal of Physical Anthropology. 2009;138: 70–81. 10.1002/ajpa.20902 18711735

[46] Redican WK. Facial Expressions in Nonhuman Primates**Supported by a National Science Foundation traineeship to the author and by National Institutes of Health Grants RR00169, HD04335, and MH22253 In: Rosenblum LA, editor. Primate Behavior. Academic Press; 1975 pp. 103–194. 10.1016/B978-0-12-534004-5.50007-5

[47] Kanazawa S. Recognition of facial expressions in a Japanese monkey (Macaca fuscata) and humans (Homo sapiens). Primates. 1996;37: 25–38. 10.1007/BF02382917

[48] Abegg C, Petit O, Thierry B. Variability in behavior frequencies and consistency in transactions across seasons in captive Japanese macaques (Macaca fuscata). Aggressive Behavior. 2003;29: 81–93. 10.1002/ab.10034

[49] Chaffin CL, Friedlen K, Waal FBMD. Dominance style of Japanese macaques compared with rhesus and stumptail macaques. American Journal of Primatology. 1995;35: 103–116. 10.1002/ajp.1350350203 31924068

[50] Petit O, Bertrand F, Thierry B. Social play in crested and japanese macaques: Testing the covariation hypothesis. Developmental Psychobiology. 2008;50: 399–407. 10.1002/dev.20305 18393281

[51] Scopa C, Palagi E. Mimic me while playing! Social tolerance and rapid facial mimicry in macaques (Macaca tonkeana and Macaca fuscata). Journal of Comparative Psychology. 2016;130: 153–161. 10.1037/com0000028 27078077

[52] Iki S, Hasegawa T. Face-to-face opening phase in Japanese macaques’ social play enhances and sustains participants’ engagement in subsequent play interaction. Anim Cogn. 2020;23: 149–158. 10.1007/s10071-019-01325-7 31720883

[53] Descovich K, Richmond SE, Leach MC, Buchanan-Smith HM, Flecknell P, Farningham DAH, et al Opportunities for refinement in neuroscience: Indicators of wellness and post-operative pain in laboratory macaques. Altex. 2019;36: 535–554. 10.14573/altex.1811061 30924506

[54] Yano M, Matsuda A, Natsume T, Ogawa S, Awaga Y, Hayashi I, et al Pain-related behavior and brain activation in cynomolgus macaques with naturally occurring endometriosis. Hum Reprod. 2019;34: 469–478. 10.1093/humrep/dey383 30597044

[55] Ogawa S, Awaga Y, Takashima M, Hama A, Matsuda A, Takamatsu H. Knee osteoarthritis pain following medial meniscectomy in the nonhuman primate. Osteoarthritis and Cartilage. 2016;24: 1190–1199. 10.1016/j.joca.2016.02.006 26944197

[56] Isa T, Yamane I, Hamai M, Inagaki H. Japanese macaques as laboratory animals. Exp Anim. 2009;58: 451–457. 10.1538/expanim.58.451 19897928

[57] Lucey P, Cohn JF, Prkachin KM, Solomon PE, Matthews I. Painful data: The UNBC-McMaster shoulder pain expression archive database Face and Gesture 2011. 2011 pp. 57–64. 10.1109/FG.2011.5771462

[58] Miyabe‐Nishiwaki T, MacIntosh AJJ, Kaneko A, Morimoto M, Suzuki J, Akari H, et al Hematological and blood chemistry values in captive Japanese macaques (Macaca fuscata fuscata). Journal of Medical Primatology. 2019;48: 338–350. 10.1111/jmp.12434 31418873

[59] Watanabe K. A Review of 50 Years of Research on the Japanese Monkeys of Koshima: Status and Dominance In: Matsuzawa T, editor. Primate Origins of Human Cognition and Behavior. Tokyo: Springer Japan; 2008 pp. 405–417. 10.1007/978-4-431-09423-4_20

[60] Koshima Field Station, Wildlife Research Center, Kyoto University. [cited 29 Jun 2020]. https://www.wrc.kyoto-u.ac.jp/koshima_st/index_e.htm

[61] Burrows AM, Waller BM, Micheletta J. Mimetic Muscles in a Despotic Macaque (Macaca mulatta) Differ from Those in a Closely Related Tolerant Macaque (M. nigra). The Anatomical Record. 2016;299: 1317–1324. 10.1002/ar.23393 27343148

[62] Hayasaka K, Fujii K, Horai S. Molecular phylogeny of macaques: implications of nucleotide sequences from an 896-base pair region of mitochondrial DNA. Mol Biol Evol. 1996;13: 1044–1053. 10.1093/oxfordjournals.molbev.a025655 8752012

[63] Burrows AM, Waller BM, Parr LA. Facial musculature in the rhesus macaque (*Macaca mulatta*): evolutionary and functional contexts with comparisons to chimpanzees and humans. Journal of Anatomy. 2009;215: 320–334. 10.1111/j.1469-7580.2009.01113.x 19563473 PMC2750044

[64] Waller BM, Vick S-J, Parr LA, Bard KA, Pasqualini MCS, Gothard KM, et al Intramuscular electrical stimulation of facial muscles in humans and chimpanzees: Duchenne revisited and extended. Emotion. 2006;6: 367–382. 10.1037/1528-3542.6.3.367 16938079 PMC2826128

[65] Waller BM, Parr LA, Gothard KM, Burrows AM, Fuglevand AJ. Mapping the contribution of single muscles to facial movements in the rhesus macaque. Physiology & Behavior. 2008;95: 93–100. 10.1016/j.physbeh.2008.05.002 18582909 PMC2637410

[66] Wexler DA. Method for unitizing protocols of descriptions of emotional states. Journal of Supplemental Abstracts Service, Catalogue of Selected Documents in Psychology, American Psychological Association. 1972;2: 116.

[67] Ekman P, Friesen WV, Hager JC. Facial Action Coding System (FACS): manual. Salt Lake City: Research Nexus; 2002.

[68] Ekman P, Friesen WV, Hager JC. FACS investigator’s guide. Salt Lake City: Research Nexus; 2002.

[69] Kawakami F, Tomonaga M, Suzuki J. The first smile: spontaneous smiles in newborn Japanese macaques (Macaca fuscata). Primates. 2017;58: 93–101. 10.1007/s10329-016-0558-7 27485748

[70] Pampush JD, Daegling DJ. The enduring puzzle of the human chin: “The Human Chin”. Evolutionary Anthropology: Issues, News, and Reviews. 2016;25: 20–35. 10.1002/evan.21471 26800015

[71] Hackley SA. Evidence for a vestigial pinna-orienting system in humans. Psychophysiology. 2015;52: 1263–1270. 10.1111/psyp.12501 26211937

[72] Guerra AB, Metzinger SE, Metzinger RC, Xie C, Xie Y, Rigby PL, et al Variability of the Postauricular Muscle Complex: Analysis of 40 Hemicadaver Dissections. Arch Facial Plast Surg. 2004;6: 342–347. 10.1001/archfaci.6.5.342 15381582

[73] Bérzin F. Occipitofrontalis muscle: functional analysis revealed by electromyography. Electromyogr Clin Neurophysiol. 1989;29: 355–358. 2689156

[74] Kushima H, Matsuo K, Yuzuriha S, Kitazawa T, Moriizumi T. The occipitofrontalis muscle is composed of two physiologically and anatomically different muscles separately affecting the positions of the eyebrow and hairline. British Journal of Plastic Surgery. 2005;58: 681–687. 10.1016/j.bjps.2005.01.006 15927153

[75] Pritchard DW, Wood MM. EMG levels in the occipitofrontalis muscles under an experimental stress condition. Biofeedback and Self-Regulation. 1983;8: 165–175. 10.1007/BF01000546 6882813

[76] Burrows AM, Li L, Waller BM, Micheletta J. Social variables exert selective pressures in the evolution and form of primate mimetic musculature. Journal of Anatomy. 2016;228: 595–607. 10.1111/joa.12440 26750637 PMC4804140

[77] Partan SR. Single and Multichannel Signal Composition: Facial Expressions and Vocalizations of Rhesus Macaques (Macaca mulatta). Behaviour. 2002;139: 993–1027.

[78] Micheletta J, Engelhardt A, Matthews L, Agil M, Waller BM. Multicomponent and Multimodal Lipsmacking in Crested Macaques (Macaca nigra). American Journal of Primatology. 2013;75: 763–773. 10.1002/ajp.22105 23225489

[79] Thierry B, Demaria C, Preuschoft S, Desportes C. Structural convergence between silent bared-teeth display and relaxed open-mouth display in the Tonkean macaque (Macaca tonkeana). Folia Primatol. 1989;52: 178–184. 10.1159/000156396 2613115

[80] Shimooka Y, Nakagawa N. Functions of an unreported “rocking-embrace” gesture between female Japanese Macaques (Macaca fuscata) in Kinkazan Island, Japan. Primates. 2014;55: 327–335. 10.1007/s10329-014-0411-9 24519610

[81] Ghazanfar AA, Chandrasekaran C, Morrill RJ. Dynamic, rhythmic facial expressions and the superior temporal sulcus of macaque monkeys: implications for the evolution of audiovisual speech. European Journal of Neuroscience. 2010;31: 1807–1817. 10.1111/j.1460-9568.2010.07209.x 20584185 PMC2898901

[82] Diogo R, Wood BA, Aziz MA, Burrows A. On the origin, homologies and evolution of primate facial muscles, with a particular focus on hominoids and a suggested unifying nomenclature for the facial muscles of the Mammalia. Journal of Anatomy. 2009;215: 300–319. 10.1111/j.1469-7580.2009.01111.x 19531159 PMC2750763

[83] Caeiro CC, Guo K, Mills DS. Dogs and humans respond to emotionally competent stimuli by producing different facial actions. Scientific Reports. 2017 10.1038/s41598-017-15091-4 29138393 PMC5686192

[84] Creanza N, Ruhlen M, Pemberton TJ, Rosenberg NA, Feldman MW, Ramachandran S. A comparison of worldwide phonemic and genetic variation in human populations. 2015 [cited 10 Jan 2020]. https://pubag.nal.usda.gov/catalog/2305116 10.1073/pnas.1424033112 PMC4321277 25605893

[85] Liebal K, Waller BM, Slocombe KE, Burrows AM. Primate Communication: A Multimodal Approach. Cambridge University Press; 2014. 10.1177/147470491301100305 PMC10480985 23864293

[86] Montag C, Panksepp J. Primal emotional-affective expressive foundations of human facial expression. Motivation and Emotion. 2016;40: 760–766. 10.1007/s11031-016-9570-x

[87] Waller BM, Whitehouse J, Micheletta J. Rethinking primate facial expression: A predictive framework. Neuroscience & Biobehavioral Reviews. 2017;82: 13–21. 10.1016/j.neubiorev.2016.09.005 27637495

[88] Murray EM, Krause WH, Stafford RJ, Bono AD, Meltzer EP, Borod JC. Asymmetry of Facial Expressions of Emotion In: Mandal MK, Awasthi A, editors. Understanding Facial Expressions in Communication: Cross-cultural and Multidisciplinary Perspectives. New Delhi: Springer India; 2015 pp. 73–99. 10.1007/978-81-322-1934-7_5

[89] Lindell AK. Continuities in Emotion Lateralization in Human and Non-Human Primates. Front Hum Neurosci. 2013;7 10.3389/fnhum.2013.00464 23964230 PMC3737467

[90] Ekman P. Darwin, deception, and facial expression. Ann N Y Acad Sci. 2003;1000: 205–221. 10.1196/annals.1280.010 14766633

[91] Yan W-J, Wu Q, Liang J, Chen Y-H, Fu X. How Fast are the Leaked Facial Expressions: The Duration of Micro-Expressions. J Nonverbal Behav. 2013;37: 217–230. 10.1007/s10919-013-0159-8

[92] Fabre-Thorpe M, Delorme A, Marlot C, Thorpe S. A Limit to the Speed of Processing in Ultra-Rapid Visual Categorization of Novel Natural Scenes. Journal of Cognitive Neuroscience. 2001;13: 171–180. 10.1162/089892901564234 11244543

[93] Kuhbandner C, Hanslmayr S, Maier MA, Pekrun R, Spitzer B, Pastötter B, et al Effects of mood on the speed of conscious perception: behavioural and electrophysiological evidence. Soc Cogn Affect Neurosci. 2009;4: 286–293. 10.1093/scan/nsp010 19351693 PMC2728630

[94] Bremhorst A, Sutter NA, Würbel H, Mills DS, Riemer S. Differences in facial expressions during positive anticipation and frustration in dogs awaiting a reward. Scientific Reports. 2019;9: 19312 10.1038/s41598-019-55714-6 31848389 PMC6917793

