# Supplementary material for: Extending the MaqFACS to measure facial movement in Japanese macaques (Macaca fuscata) reveals a wide repertoire potential
Source: PLoS One. 2021 Jan 7;16(1):e0245117. doi: 10.1371/journal.pone.0245117 (PMC7790396; doi:10.1371/journal.pone.0245117)
Supplement: S1 Text — (DOCX) [file pone.0245117.s001.docx]

**S1 Text for:**

Extending the MaqFACS to measure facial movement in Japanese macaques (*Macaca fuscata*) reveals a wide repertoire potential

Catia Correia Caeiro^1*^, Kathryn Holmes^2^, Takako Miyabe-Nishiwaki^1^

^1^Primate Research Institute, Kyoto University, Inuyama, Aichi, Japan

^2^School of Psychology, University of Lincoln, Lincoln, Lincolnshire, United Kingdom

***Corresponding author**

E-mail: catia_caeiro@hotmail.com (CC)

**ORCID**: https://orcid.org/0000-0002-2819-6039

**Methodology**

**Subjects and data collection**

**Observational field experiments:** these field experiments were conducted at the Koshima research site by postgraduate students and researchers from KUPRI and WRC. All studies were independent and unrelated to the present study. These experiments consisted on a range of cognitive and behavioural tasks, where the macaques could choose to participate (or not). The tasks were setup on the beach at Koshima and researchers would wait until individuals decided to perform the tasks to collect observational data (e.g. choices on a task, looking times, behavioural responses). Examples of these tasks are: 1) visual paired comparison of images; 2) odour discrimination; 3) response to different types of music; 4) taste preferences; 5) visual patterns discrimination, among others. For the current study, we collected ad libitum footage of the macaques participating in these tasks at a distance of minimum 2m (both to not disturb the experiments and to capture naturalistic responses during the tasks). We also filmed the macaques when they were not participating in the experiments, in order to try to include a variety of contexts, as we did for the groups living at KUPRI (e.g. solitary, conspecific, agonistic, affiliative, resting, grooming, foraging/feeding, sexual, play, and human interaction behaviours, among others). For FACS development it is important to capture a wide variety of contexts to ensure the full potentiality for facial movement is captured and experimental tasks provide a proxy for naturalistic behaviours.

**Rational for target sample:** FACS are tools based on the underlying anatomy and observable appearance changes (i.e. it links what we *see on the face* to what caused the movement) of a particular species. Faces as a global object are very constant (even between species, e.g. eyes above nose and above mouth), but the arrangement of these features between species is highly variable (shape, size, colour, inter-feature distance, etc.). Within a species, these tend to form a constant pattern with fixed characteristics, but still with some variation between populations and individuals (e.g. amount of fur surrounding face, craniometry factors). Hence, it is important to make a distinction from the sample collected in this work, in contrast to typical samples collected for other empirical studies (e.g. cognitive or ecological testing). For our current work, one of the main goals when collecting footage was to document the ***potentiality*** for movement on this species face (i.e. given the number, position, size, etc of muscles and the visible changes on the face during movement), and **not** what the typical movements of the species are or how frequent they might be. Nonetheless, these and other quantifiable variables can be measured with FACS. Therefore, the important factors we accounted for in order to ensure a representative sample were: 1) diversity of populations/sub-species (e.g. Koshima vs Jigokudani), 2) diversity of morpho-types (e.g. long lighter fur vs shorter darker fur), 3) diversity in age and sex (which naturally existed in all groups sampled), 4) diversity of living environments (e.g. hot springs mountains vs beach on an island), 5) diversity of behaviours performed and contexts (including non-social, affiliative, agonistic, and neutral, etc.). Since we had access to a very large sample (N>250), the priority when collecting footage was to try to sample always a different individual during focals (around 1min), but if interesting behaviours were observed in an individual that was not the focal (e.g. focal individual was grooming, and we had collected plenty of grooming focals before, the focal would be stopped and ad libitum footage collected with more rare behaviours, such as sexual behaviours). Therefore, the footage collected by the authors for the present work (~20h) was obtained by spending around 20h observing the different groups of Japanese macaques, filming continuously and switching between subjects to get the greatest possible variety of facial movements in the given time.
